# The Mechanics of Tumor Cells Dictate Malignancy via Cytoskeleton-Mediated APC/Wnt/β-Catenin Signaling

**DOI:** 10.34133/research.0224

**Published:** 2023-09-21

**Authors:** Xi Chen, Zichen Xu, Kai Tang, Guanshuo Hu, Pengyu Du, Junfang Wang, Cunyu Zhang, Ying Xin, Keming Li, Qiantang Zhang, Jianjun Hu, Zhuxue Zhang, Mo Yang, Guixue Wang, Youhua Tan

**Affiliations:** ^1^ The Hong Kong Polytechnic University Shenzhen Research Institute, Shenzhen, 518057, China.; ^2^ Key Laboratory for Biorheological Science and Technology of Ministry of Education, State and Local Joint Engineering Laboratory for Vascular Implants, Bioengineering College of Chongqing University, Chongqing, 400030, China.; ^3^ Research Institute of Smart Ageing, The Hong Kong Polytechnic University, Hong Kong, China.; ^4^ Department of Biomedical Engineering, The Hong Kong Polytechnic University, Hong Kong, China.; ^5^ Department of Pathology, Guizhou Provincial People's Hospital, Guiyang, Guizhou, 550002, China.

## Abstract

Tumor cells progressively remodel cytoskeletal structures and reduce cellular stiffness during tumor progression, implicating the correlation between cell mechanics and malignancy. However, the roles of tumor cell cytoskeleton and the mechanics in tumor progression remain incompletely understood. We report that softening/stiffening tumor cells by targeting actomyosin promotes/suppresses self-renewal in vitro and tumorigenic potential in vivo. Weakening/strengthening actin cytoskeleton impairs/reinforces the interaction between adenomatous polyposis coli (APC) and β-catenin, which facilitates β-catenin nuclear/cytoplasmic localization. Nuclear β-catenin binds to the promoter of Oct4, which enhances its transcription that is crucial in sustaining self-renewal and malignancy. These results demonstrate that the mechanics of tumor cells dictate self-renewal through cytoskeleton–APC–Wnt/β-catenin–Oct4 signaling, which are correlated with tumor differentiation and patient survival. This study unveils an uncovered regulatory role of cell mechanics in self-renewal and malignancy, and identifies tumor cell mechanics as a hallmark not only for cancer diagnosis but also for mechanotargeting.

## Introduction

Many solid tumors are believed to be organized as a hierarchy with cancer stem-like cells (CSCs) at the apex, a rare and tumorigenic subpopulation of cancer cells that possess the ability to self-renew and drive tumorigenesis and metastasis [1]. Self-renewal is critical in sustaining long-term tumor propagation, dissemination, and relapse. Therefore, understanding the mechanisms underlying tumor cell self-renewal is essential for the development of novel therapeutic strategies for effective treatment.

Except many important biochemical factors [1], cancer cells, especially CSCs, exhibit unique mechanical properties [2–4], including the reduction of cytoskeletal structures and cellular stiffness in many types of cancer. Low cell stiffness is correlated with tumor malignancy and poor clinical outcome [2,5]. For example, the elastic modulus of healthy human bladder cells is one order of magnitude higher than that of cancer cells [6]. Cell stiffness progressively decreases when human urothelial and breast epithelial cells are transformed from normal, to noninvasive, and to invasive phenotype [7,8]. In clinical specimens, the stiffness of hepatocellular carcinoma (HCC) cells is inversely associated with their malignant potential [9]. Metastatic tumor cells from patients with lung, breast, and pancreas cancer show ~70% lower stiffness than benign cells from the same patients [10]. Mechanical stiffness of primary ovarian tumor cells can grade their malignant potential [11]. Importantly, CSCs with the enhanced ability to self-renew are much softer than conventional tumor cells in multiple types of cancer, such as skin [12], breast [13], and lung cancer [14]. Recent evidence shows that the sorted soft subpopulation of cancer cells exhibit higher self-renewal and tumorigenicity than do stiff cells [15,16] and resist cytotoxic T cell-mediated killing [17,18]. All these findings suggest the inverse correlation between tumor cell mechanics and malignancy or self-renewal.

The mechanics of a cell are mainly governed by its cytoskeletal structure and the associated proteins. Indeed, considerable changes in cytoskeleton are associated with malignant transformation and tumor progression and can lead to the altered tumor cell mechanics [19,20]. The cytoskeleton interacts with various signaling molecules, including the well-known tumor suppressor adenomatous polyposis coli (APC), to influence cellular functions. APC associates with actin and microtubule through the basic domain and interacts with β-catenin together with the tumor suppressor Axin and the Ser/Thr kinases glycogen synthase kinase-3 (GSK-3) in the destruction complex within the cytoplasm [21,22]. The dissociation of β-catenin from APC facilitates its nuclear translocation to activate Wnt/β-catenin signaling, which is critical in the maintenance of self-renewal. Since the mechanics of cytoskeleton are crucial in multiple cellular functions [23,24], we thus hypothesize the existence of an intrinsic link between tumor cell mechanics and self-renewal via cytoskeleton–APC-mediated Wnt/β-catenin signaling.

## Results

### Softening tumor cells promotes self-renewal and tumorigenicity

In this study, we utilized HCC as a model of disease and first explored the relationship between tumor cell mechanics and self-renewal. Liver CSCs were selected based on the surface marker EpCAM [25] and by 3-dimensional (3D) soft fibrin [12,26], respectively. Tumor cell self-renewal was assessed by colony formation in soft agar and sphere formation assay (SFA) and by colony growth in soft fibrin. EpCAM^+^ cells grew much larger tumor spheroids in soft fibrin and more colonies in soft agar than did EpCAM^−^ cells and control cells, while EpCAM^−^ cells generated the smallest spheroids and the least number of colonies (Fig. S1A to C), suggesting the high self-renewal of EpCAM^+^ cells. Notably, EpCAM^+^ cells (~1 kPa) exhibited much lower levels of F-actin and stiffness than did control and EpCAM^−^ cells (~3 kPa) (Fig. S1D to F). In addition, fibrin-selected CSCs up-regulated multiple self-renewal markers (Fig. S1G and H) and exhibited lower stiffness (1.5 versus 3.0 kPa) and F-actin than did control cells (Fig. S1I to K). Interestingly, both EpCAM^+^ cells and fibrin-selected CSCs generated higher contractile forces than did EpCAM^−^ cells and control cells (Fig. S1L and M). These results suggest the correlation between tumor cell mechanics and self-renewal in HCC.

**Fig. 1.**
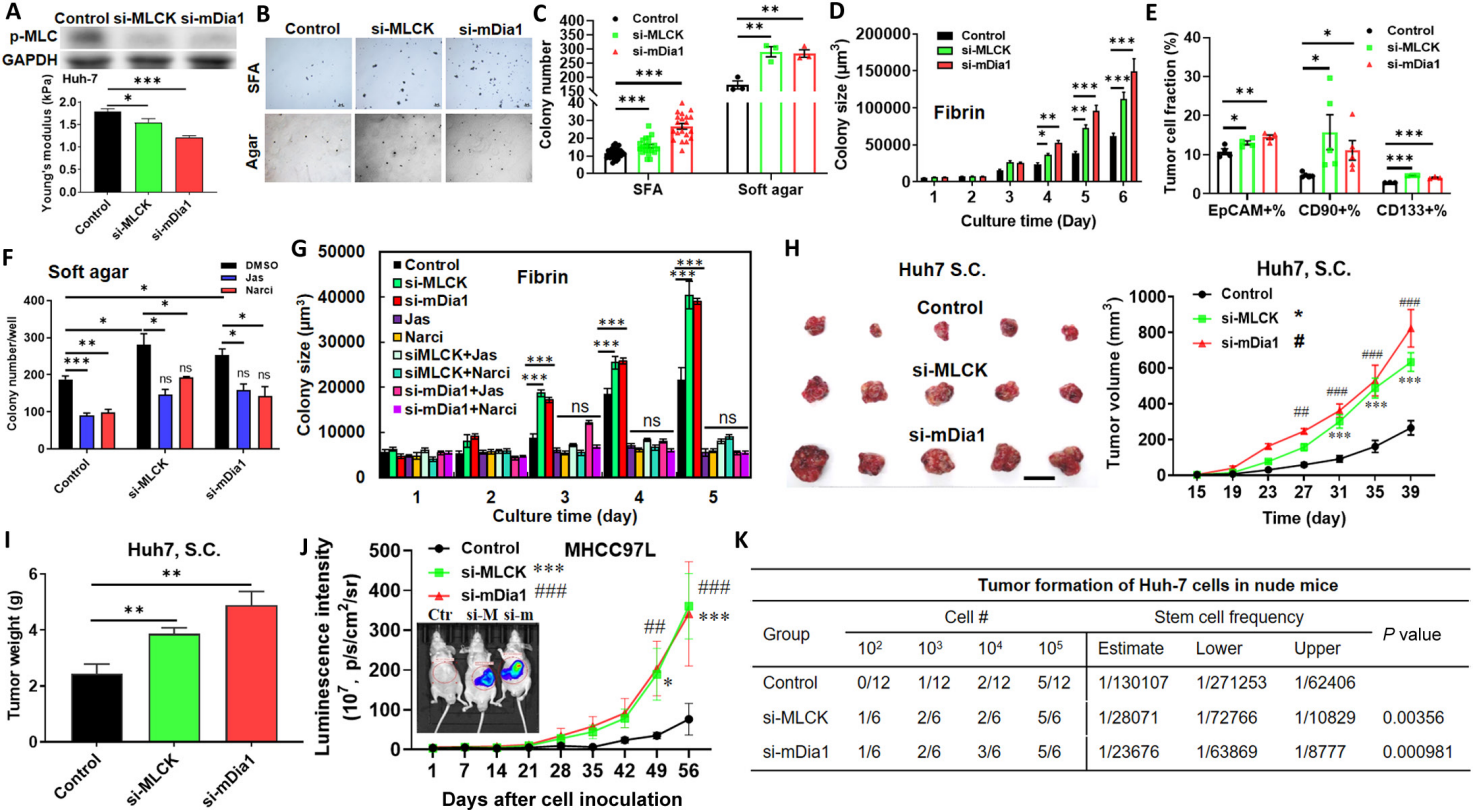
Softening cells promotes self-renewal and tumorigenicity. (A) Phosphorylation of MLC and cell stiffness after knocking down MLCK and mDia1 using 10 nM siRNAs. *n* > 80 cells. (B and C) Formation of tumor spheroids in sphere formation assay (SFA) and soft agar assay after HCC cells were softened. *n* = 3 and 20 for soft agar assay and SFA, respectively. (D) Growth of tumor spheroids generated by softened tumor cells in soft fibrin. Representative of 3 independent experiments. (E) Fractions of EpCAM^+^, CD90^+^, and CD133^+^ HCC cells. *n* = 4, 5, and 3 for EpCAM, CD90, and CD133, respectively. (F and G) Colony formation and growth in soft agar and soft fibrin after Huh-7 cells were transfected with the siRNAs of MLCK and mDia1 and then treated with 20 nM jasplakinolide (Jas) or 1 nM narciclasine (Narci). *n* = 3. (H and I) Tumor growth after the softened Huh-7 cells were inoculated subcutaneously (S.C.) into the flank of nude mice. *n* = 5 for each condition. After the experiment, the tumor tissues were retrieved and weighted. Scale bar, 1 cm. (J) Tumor growth after the softened MHCC97L cells were prelabeled with luciferase and then inoculated into the liver of nude mice. *n* = 13 for each condition. (K) Tumor incidence of the softened tumor cells in the limiting dilution assay. In total, 100, 1,000, 10,000, and 100,000 of control and softened Huh-7 cells were inoculated subcutaneously into the flank of nude mice. The tumor incidence was measured at week 8. The stem cell frequency and *P* value (chi-square test) were calculated using the tools at http://bioinf.wehi.edu.au/software/elda/. Mean ± SEM. Analysis of variance (ANOVA) was used for the statistical analysis together with the post hoc Bonferroni test. *P < 0.05; **P < 0.01; ***P < 0.001; ^#^*P* < 0.05; ^##^*P* < 0.01; ^###^*P* < 0.001. * or #, the comparison between “si-MLCK” or “si-mDia1” and “Control”. ns: no significance.

To dissect the role of cell mechanics in self-renewal, the mechanical properties of tumor cells were modulated by targeting actomyosin activity [27]. Huh-7 cells were softened by silencing myosin light chain kinase (MLCK) and mammalian diaphanous-related formin 1 (mDia1) (Fig. S12A), which regulates myosin activity and F-actin polymerization, respectively. Such treatment decreased F-actin by 18 to 29% (Fig. S2B), phosphorylation of myosin light chain (MLC), contractile forces by 25 to 32% (Fig. S3G), and cellular stiffness by 13 to 32% (Fig. 1A) but did not affect cell area, morphology, microtubule, vimentin, and vinculin (Fig. S3A to F). Strikingly, moderately silencing MLCK or mDia1 considerably enhanced the colony formation in SFA and soft agar by 35 to 130% and ~65% (Fig. 1B and C), promoted the growth of tumor spheroids in soft fibrin by 80 to 140% (Fig. 1D), and elevated the fractions of EpCAM^+^, CD90^+^, and CD133^+^ cells by 20 to 34%, 130 to 230%, and 46 to 64%, respectively (Fig. 1E), all of which labeled liver CSCs [25,28,29]. Further, softened cells up-regulated the self-renewal markers (Fig. S4A and B) and had no promotive effect on cell survival and proliferation (Fig. S2C and D). CSCs exhibited decreased stiffness but elevated contractility and self-renewal (Fig. S1). Inhibiting actomyosin reduced both cell stiffness and contractility while enhancing self-renewal (Fig. S3G), suggesting that the promotive effect of actomyosin activity on self-renewal might be not through the influence on cell contractility but rather on cellular stiffness. We also found that softening cells enhanced the formation and growth of tumor spheroids and the percentage of EpCAM^+^ and CD90^+^ cells in several other HCC cells (Hep3B, HepG2, MHCC97L) (Fig. S4C to J). To further strengthen this finding, we knocked down another actin filament crosslinker, α-actinin [30] (Fig. S5A), which markedly reduced F-actin and cellular stiffness (Fig. S5B and C). Silencing α-actinin elevated the growth of tumor spheroids in soft fibrin by 300% and colony formation in soft agar by 95% (Fig. S15D and E) and up-regulated the self-renewal markers (Fig. S5F to H).

Further, HCC cells were also softened by actin polymerization inhibitor cytochalasin D, Rho-associated protein kinase (ROCK) inhibitor Y-27632, and myosin inhibitor blebbistatin. The treatment by low doses of these drugs moderately reduced F-actin and cell stiffness (Figs. S2E and S6G) and significantly increased the formation and growth of tumor spheroids and the EpCAM^+^ subpopulation (Fig. S4K to N). This effect was not due to the influence on cell proliferation (Fig. S2F). We further tested the influence of different levels of cell softening on self-renewal. Moderately softening cells using low doses of siRNAs or inhibitors decreased cell stiffness by 13 to 34%, while high doses softened cells by 53 to 84% (Fig. S6A to D and G). Moderate cell softening facilitated the formation and growth of tumor spheroids, while considerable softening suppressed self-renewal (Fig. S6E, F, H, and I). This might be due to that considerable but not moderate softening inhibited cell proliferation (Figs. S2C, D, and F and S6J to L). Interestingly, pharmacologically disrupting microtubules or intermediate filament protein vimentin at low doses had no effect on cell proliferation but significantly suppressed colony formation and tumor spheroid growth (Fig. S7). These results suggest that moderately softening actin cytoskeleton promotes tumor cell self-renewal.

To test whether the effect of cell mechanics on self-renewal is reversible, HCC cells were softened and then stiffened by actin polymerization inducer jasplakinolide or Rho activator narciclasine. Softening tumor cells increased the colony formation and growth, while concurrent stiffening reduced these abilities to the similar or even lower level as control cells (Fig. 1F and G). In addition, moderately softening cells also promoted tumor cell self-renewal in breast, cervical, colon, and lung cancer (Fig. S8A to D), suggesting that moderate cell softening promotes self-renewal in multiple types of cancer.

We further examined the influence of cell mechanics on tumorigenicity. Softening Huh-7 cells remarkably promoted the tumor growth by 130 to 210% and tumor weight by 58 to 100% in the subcutaneous xenograft model (Fig. 1H and I). This effect was further examined in an orthotopic tumor model. MHCC97L cells were prelabeled with luciferase and then inoculated into the liver of nude mice. Compared to the control group, softening cells considerably promoted tumor growth in the liver by over 350% (Fig. 1J). To test the effect on tumor formation, we conducted limiting dilution assay by inoculating 100, 1,000, 10,000, and 100,000 softened tumor cells subcutaneously into nude mice. The results show that softening cells greatly enhanced the tumor incidence rate and elevated the estimated stem cell frequency by >5-fold (Fig. 1K). Collectively, these results suggest that moderately softening cells is sufficient to promote tumorigenicity.

### Stiffening tumor cells suppresses self-renewal and tumorigenicity

**Fig. 2.**
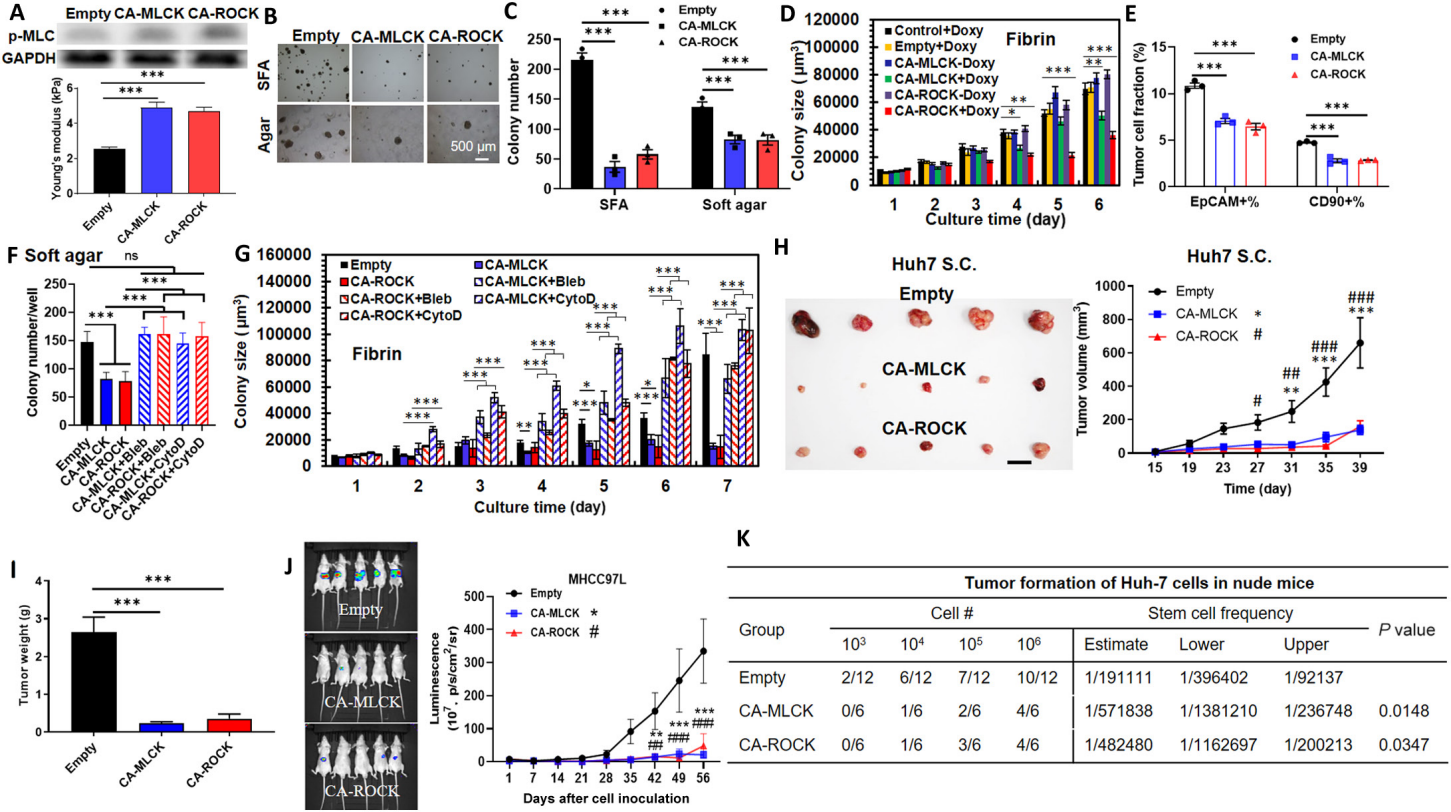
Stiffening cells suppresses self-renewal and tumorigenicity. (A) Phosphorylation of MLC and cell stiffness after expressing the constitutive active (CA) forms of MLCK and ROCK mutants. *n* > 100 cells. (B and C) Formation of tumor spheroids generated by the stiffened tumor cells in SFA and soft agar. *n* = 3. (D) Growth of tumor spheroids in soft agar with or without the induction of doxycycline (Doxy). Representative of 3 independent experiments. (E) Fractions of EpCAM^+^ and CD90^+^ subpopulations. *n* = 3 for both EpCAM and CD90. (F and G) Colony formation and growth in soft agar and soft fibrin after Huh-7 cells were transfected with CA-MLCK or CA-ROCK plasmids and then treated with 6 μM blebbistatin (Bleb) or 0.1 μM cytochalasin D (CytoD). *n* = 3. (H and I) Tumor growth after stiffened Huh-7 cells inoculated subcutaneously (S.C.) into the flank of nude mice. *n* = 5 for each condition. After the experiment, the tumor tissues were retrieved and weighed. Scale bar, 1 cm. (J) Tumor growth after the stiffened MHCC97L cells were prelabeled with luciferase and then inoculated into the liver of nude mice. *n* = 13 for each condition. (K) Tumor incidence of the stiffened tumor cells in the limiting dilution assay. In total, 1,000, 10,000, 100,000, and 1,000,000 of control and stiffened Huh-7 cells were inoculated subcutaneously into the flank of nude mice. The tumor incidence was measured at week 8. The stem cell frequency and *P* value (chi-square test) were calculated using the tools at http://bioinf.wehi.edu.au/software/elda/. Mean ± SEM. ANOVA was used for the statistical analysis together with the post hoc Bonferroni test. *P < 0.05; **P < 0.01; ***P < 0.001; ^#^*P* < 0.05; ^##^*P* < 0.01; ^###^*P* < 0.001. * or #, the comparison between “CA-MLCK” or “CA-ROCK” and “Empty”.

We next explored the necessity of low cell mechanics in sustaining tumor cell self-renewal. Toward this goal, Huh-7 cells were transfected with doxycycline-inducible constitutive active (CA) form of MLCK or ROCK [31], which enhanced the amount of F-actin by ~13% (Fig. S2G), phosphorylation of MLC, contractile forces by 49 to 118% (Fig. S3N), and cellular stiffness by ~90% (Fig. 2A). Stiffening cells substantially suppressed colony formation in SFA by 73 to 83% and in soft agar by ~40%, and reduced the spheroid growth in soft fibrin by 54 to 70% (Fig. 2B to D). The plasmid itself had no influence on colony growth without the induction of doxycycline (Fig. 2D). Further, stiffening cells reduced the percentages of EpCAM^+^ and CD90^+^ subpopulation by ~40% (Fig. 2E), and down-regulated multiple self-renewal markers (Fig. S9A and B). The suppressive effect of cell stiffening on self-renewal was also observed in other HCC cells (MHCC97L and PLC/PRF/5) and CSCs (Fig. S9C to F). In addition, pharmacologic treatment of HCC cells and CSCs using jasplakinolide and narciclasine significantly increased F-actin and cell stiffness (Figs. S2I and S9G) and suppressed the colony formation and growth, EpCAM^+^ fraction, and the expressions of self-renewal genes (Fig. S9H to L).

We next examined the reversibility of the stiffening effect on self-renewal. Huh-7 cells were transfected with CA-ROCK or CA-MLCK plasmids and then treated with blebbistatin and cytochalasin D. Stiffening cells greatly suppressed the colony formation and growth, while concurrent softening rescued these abilities to the level of control cells (Fig. 2F and G). Further, control cells, CA-ROCK/CA-MLCK Huh-7, and CSCs without the induction of doxycycline generated the largest tumor spheroids, while the cells with the induction formed the smallest spheroids (Fig. S10). CA-ROCK/CA-MLCK cells without/with doxycycline for the first 6 days generated similar/smaller sizes of tumor spheroids compared to control cells, the growth of which was notably inhibited/enhanced after the addition/removal of doxycycline (Fig. S10). These findings suggest that the influence of cell mechanics on tumor cell self-renewal is reversible. It was noted that stiffening cells had no significant effect on cell area, morphology, microtubule, vimentin, and vinculin (Fig. S3H to M) and did not suppress or even promoted cell proliferation (Fig. S12H and J), which could not explain the suppressive effect on self-renewal. In addition, stiffening cells also inhibited the self-renewal in breast, cervical, colon, and lung cancer (Fig. S8E to H). In summary, these findings demonstrate that stiffening cells suppresses tumor cell self-renewal.

We further examined the role of low cell stiffness in tumor formation in vivo. Stiffened Huh-7 cells were inoculated subcutaneously into nude mice. Cell stiffening reduced the tumor size and weight by over 75% and 85% compared to the empty group (Fig. 2H and I), respectively. Further, stiffened MHCC97L cells with prelabeled luciferase were inoculated into the liver of nude mice to develop the orthotopic tumor model. Stiffening cells suppressed the growth of liver tumors by 85 to 94% at day 56 (Fig. 2J). Limiting dilution assay was conducted to test the influence of cell stiffening on tumor incidence. The results show that stiffening tumor cells remarkably reduced the tumor incidence rate and decreased the estimated stem cell frequency within the tumor cell population by over 60% (Fig. 2K). All these results demonstrate that low cell stiffness is required to sustain tumor cell self-renewal and tumorigenicity.

### Actomyosin-mediated cellular mechanics drive self-renewal through Wnt/β-catenin signaling

**Fig. 3.**
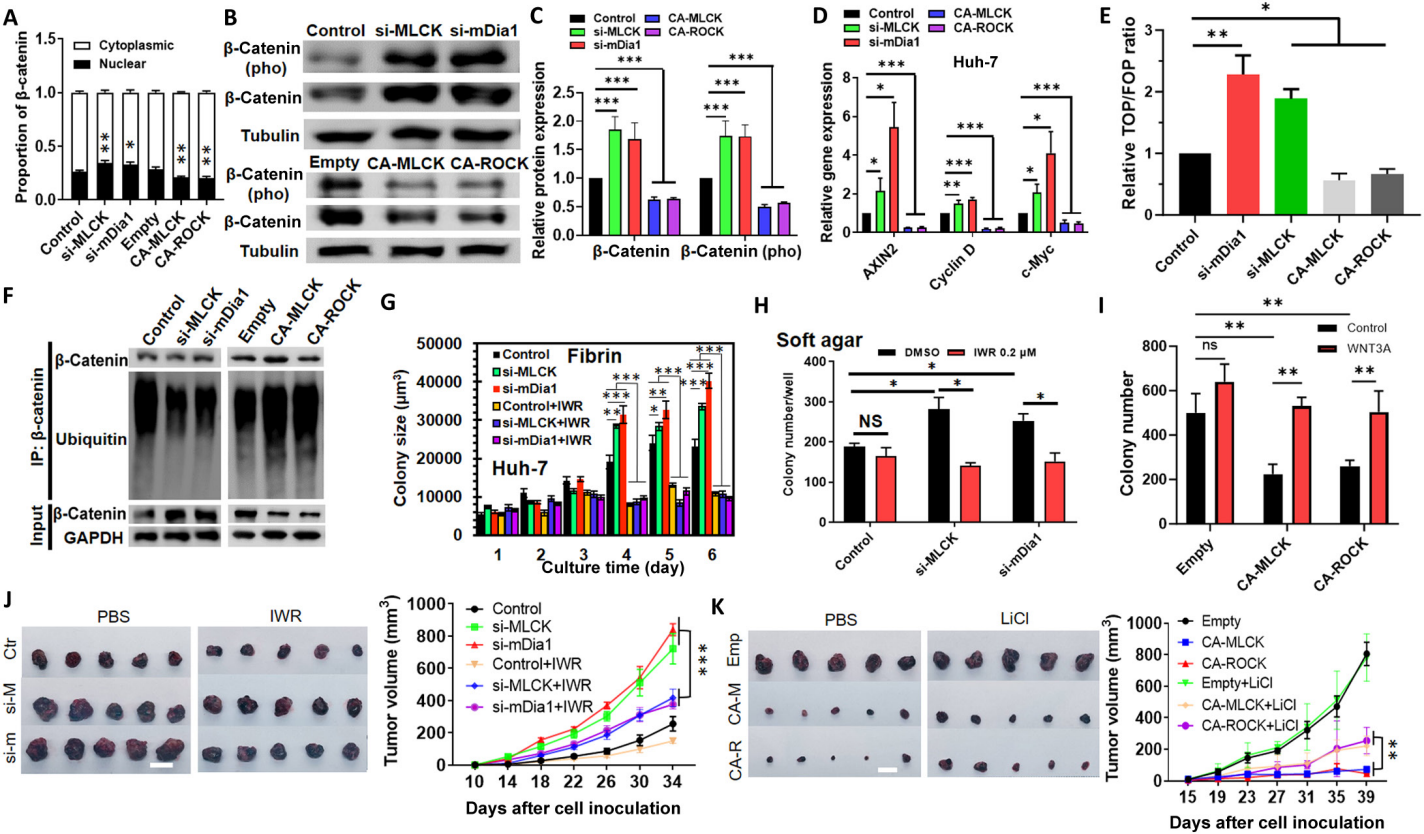
Cell mechanics regulate self-renewal and tumorigenicity through Wnt/β-catenin signaling. (A) Subcellular localization of β-catenin in softened and stiffened tumor cells. *n* > 60 cells. (B and C) Immunoblotting of the total and phosphorylated β-catenin. The representative images were shown in (B), and the results were quantified in (C). *n* = 3. (D) Expressions of Wnt downstream genes analyzed by qRT-PCR. *n* = 3. (E) Cell mechanics affect the TOP/FOP ratio representative of TCF luciferase reporter activity (β-catenin activity). Huh-7 cells were transfected with the β-catenin reporter plasmid and the mutant control, while the mechanics of tumor cells were modulated as indicated. The TOP/FOP ratio was measured. *n* = 3. (F) β-Catenin ubiquitination in softened and stiffened tumor cells. Representative of 2 independent experiments. (G and H) Formation and growth of tumor spheroids in soft agar and fibrin after Huh-7 cells were softened and then treated with Wnt inhibitor IWR. *n* = 3. (I) Colony formation in soft agar after Huh-7 cells were stiffened and then treated with Wnt ligand Wnt3A. *n* = 3. (J and K) Growth of xenografts in the subcutaneous model. Huh-7 cells were transfected with the siRNAs of MLCK and mDia1 or CA-MLCK and CA-ROCK plasmids and then inoculated subcutaneously into the flank of nude mice. *n* = 10. After 10 or 15 days, the mice in each condition were randomly separated into 2 groups, which were treated with saline, IWR (25 mmol/kg/day), or LiCl (10 mmol/kg/day). *n* = 5 for each condition. The tumor size was measured at the indicated time points. Scale bar, 1 cm. Mean ± SEM. ANOVA was used for the statistical analysis together with the post hoc Bonferroni test. *P < 0.05; **P < 0.01; ***P < 0.001.

We next investigated the mechanism by which cell mechanics drive self-renewal. RNA sequencing was conducted in softened tumor cells to identify the differentially regulated signaling pathways. The bioinformatic analysis showed that the pathways related to Wnt/β-catenin signaling and stemness were significantly enriched and activated (Fig. S11), suggesting their potential roles in cell mechanics-induced self-renewal. Wnt/β-catenin signaling is crucial in tumor progression [32] and interacts with actin cytoskeleton to regulate various cellular functions, including self-renewal [33]. To activate this pathway, β-catenin dissociates from the destruction complex in the cytoplasm and translocates into the nucleus to initiate the transcription of downstream genes [34]. To dissect its role, the influence of cell mechanics on Wnt/β-catenin signaling was examined. Softening/stiffening cells promoted/suppressed the translocation of β-catenin into the nucleus (Fig. 3A), the total and phosphorylated β-catenin (Fig. 3B and C and Fig. S12C), the expressions of Wnt downstream genes (Fig. 3D and Fig. S12A and B), and the activity of β-catenin (Fig. 3E). The association of cytoplasmic β-catenin with the destruction complex leads to its ubiquitination and proteolysis. To test this possibility, we immunoprecipitated β-catenin from lysates of the treated cells and found that softening/stiffening cells reduced/enhanced the ubiquitination of β-catenin (Fig. 3F), which might further lead to the activation/inhibition of Wnt/β-catenin signaling. These findings demonstrate that cell mechanics influence the Wnt/β-catenin activation.

To test its role in cell mechanics-regulated self-renewal, Huh-7 cells were softened/stiffened and then treated with Wnt inhibitor IWR-1-endo (IWR)/ligand Wnt3A or activator LiCl. Softening/stiffening cells promoted/inhibited the formation and growth of tumor spheroids and the expressions of self-renewal genes, while concurrently inhibiting/activating Wnt abrogated this effect and restored tumor cell self-renewal to the level of control cells (Fig. 3G to I and Fig. S12D to H, K, and L). Inhibiting Wnt pathway suppressed colony growth but not formation (Fig. 3G and H and Fig. S12D), reduced the EpCAM^+^ fraction (Fig. S12I), but did not affect self-renewal genes (Fig. S12K), while activating it did not influence colony formation and growth, EpCAM^+^ fraction, or self-renewal genes (Fig. 3H and I and Fig. S12E to H and J). The pharmacologic treatment had no detectable influence on tumor cell survival (Fig. S12J). We next examined the influence of Wnt/β-catenin signaling on cell mechanics-mediated tumorigenicity. Softened/stiffened Huh-7 cells were inoculated subcutaneously into nude mice, which were treated with IWR/LiCl. Softening cells promoted tumor growth, while concurrently inhibiting Wnt diminished this effect (Fig. 3J). On the other hand, stiffening cells suppressed tumor growth, while activating Wnt partially reversed this effect (Fig. 3K). All these results conclude that actomyosin-mediated cell mechanics regulate self-renewal and tumorigenicity through Wnt/β-catenin signaling.

### Cell mechanics influence the interconversion between non-CSCs and CSCs in a Wnt-dependent manner

Within a heterogeneous tumor, CSCs differentiate into non-CSCs, while non-CSCs can dedifferentiate into CSCs under certain conditions. To elucidate the roles of cell mechanics in these processes, we first investigated the alteration of cell stiffness during CSC differentiation. Fibrin-selected CSCs (~1.5 kPa) exhibited lower stiffness than non-CSCs (~2.9 kPa) and increased their stiffness (2.3 kPa) after 7-day culture on glass (Fig. S13A), which is consistent with our earlier finding in melanoma CSCs [26]. This treatment reduced the EpCAM^+^ fraction by ~48% and spheroid growth by ~70% to the levels of non-CSCs and decreased the colony formation and CD133 expression by ~80% and 36% (Fig. S13B to E), suggesting the differentiation or loss of self-renewal in CSCs. Importantly, inhibiting ROCK prevented the increase in cellular stiffness (Fig. S13A), fully restored the EpCAM^+^ fraction and CD133 expression to the levels of CSCs, and partially rescued the colony formation and growth (Fig. S13B to E). These findings suggest that the increase of cell stiffness associates with CSC differentiation, which can be prevented by cell softening.

We further explored the influence of cell mechanics on the interconversion between CSCs and non-CSCs. Softening EpCAM^−^ cells (non-CSCs) increased the EpCAM^+^ fraction (CSCs) by 40 to 84% (Fig. S14A) and the formation and growth of tumor spheroids by 150 to 250% (Fig. S14B) and 230 to 300% (Fig. S14C), respectively. On the other hand, stiffening EpCAM^+^ cells reduced the EpCAM^+^ fraction by 40 to 58% (Fig. S14D) and the colony formation and growth by 58 to 89% (Fig. S14E) and 58 to 65% (Fig. S14F), respectively. However, stiffening EpCAM^−^ cells and softening EpCAM^+^ cells had no significant effect on the EpCAM^+^ fraction and tumor cell self-renewal (Fig. S14M to P). These results suggest that cell softening facilitates the dedifferentiation of non-CSCs into CSCs, while cell stiffening promotes CSC differentiation. To elucidate the underlying mechanism, EpCAM^−^ cells were softened in the presence of Wnt inhibitor. Concurrently inhibiting Wnt signaling diminished the promotive effect of cell softening on the self-renewal of EpCAM^−^ cells and reduced the ability to a level even lower than that of control (Fig. S14G to I), while activating Wnt in the stiffened EpCAM^+^ cells restored the self-renewal ability to the control level (Fig. S14J to L). All these results suggest that cell mechanics regulate the Wnt-dependent interconversion between CSCs and non-CSCs.

### The mechanics of actin cytoskeleton regulate Wnt/β-catenin signaling through the interaction with APC

**Fig. 4.**
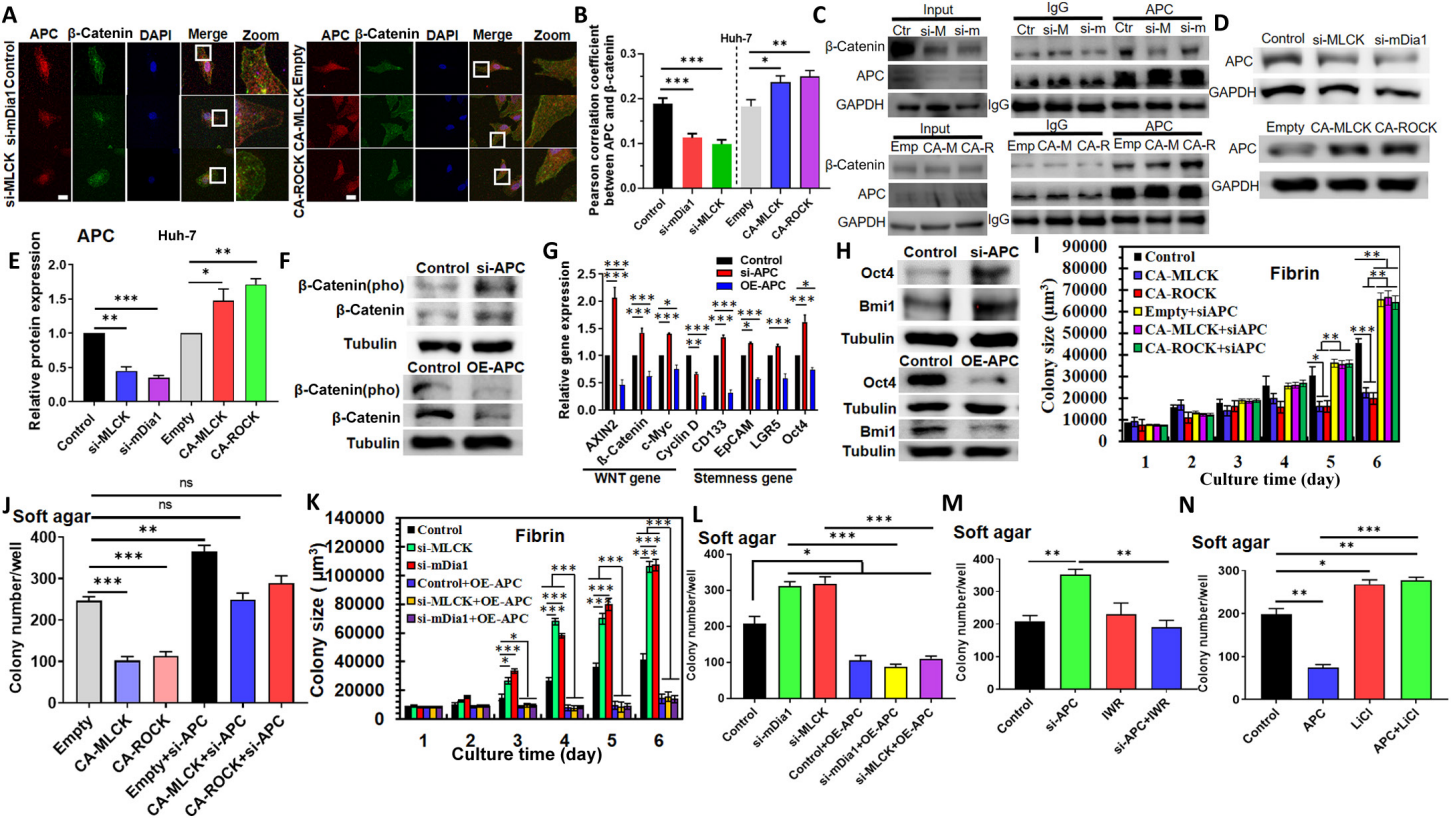
Cell mechanics regulate Wnt signaling and self-renewal through APC–β-catenin interaction. (A and B) Colocalization between APC and β-catenin stained by immunofluorescence in softened and stiffened tumor cells. The representative images were shown in (A), and the Pearson correlation coefficient was calculated in (B). *n* = 30 cells per condition. Scale bar, 25 μm. (C) Coimmunoprecipitation analysis of the interaction between APC and β-catenin. Representative of 2 independent experiments. (D and E) Immunoblotting of APC in softened and stiffened tumor cells. *n* = 3. (F) Immunoblotting of the total and phosphorylated β-catenin when APC was silenced or overexpressed. Representative of 2 independent experiments. (G) Expressions of Wnt downstream genes and stemness genes measured by qRT-PCR. *n* = 3. (H) Expressions of Oct4 and Bmi1 measured by Western blotting. Representative of 2 independent experiments. (I and J) Formation and growth of tumor spheroids in soft agar and fibrin when stiffened Huh-7 cells were transfected with APC siRNA. *n* = 3. (K and L) Formation and growth of tumor spheroids in soft agar and fibrin when softened cells were transfected with APC plasmids. *n* = 3. (M and N) Formation of tumor spheroids in soft agar when Huh-7 cells were transfected with APC siRNA or plasmids and then treated with IWR or LiCl. *n* = 3. ANOVA was used for the statistical analysis together with the post hoc Bonferroni test. *P < 0.05; **P < 0.01; ***P < 0.001.

We next dissected how cell mechanics regulated Wnt-dependent self-renewal. APC can interact with actin and microtubule directly through the basic domain and indirectly through other proteins [33], which may influence the binding between APC and β-catenin and eventually affect the Wnt/β-catenin signaling [34]. We thus hypothesized that the mechanics of actin cytoskeleton affected Wnt/β-catenin signaling via APC. To test this idea, we first examined the influence of cell mechanics on APC–β-catenin interaction. Softening/stiffening cells decreased/increased the colocalization between APC and β-catenin (Fig. 4A and B). Further, coimmunoprecipitation analysis showed that softening cells decreased APC and weakened the binding between β-catenin and APC (Fig. 4C to E and Fig. S15A, B, and D), while stiffening cells increased APC and strengthened this interaction (Fig. 4C to E and Fig. S15A to C). In addition, soft CSCs exhibited reduced APC expression (Fig. S15E). These findings suggest that the mechanics of actin cytoskeleton regulate APC–β-catenin interaction. Interestingly, weakening/strengthening cytoskeleton reduced/enhanced the colocalization between APC and F-actin (Fig. S16A and B). α-Catenin interacts with APC and stabilizes the association between APC and β-catenin, leading to β-catenin proteolysis [35]. Softening/stiffening cells down-/up-regulated α-catenin (Fig. S16C and D). The influence of cell mechanics on APC–F-actin interaction and α-catenin may affect APC–β-catenin interaction. The mechanism by which cell mechanics impact the association between APC and β-catenin requires further investigation.

APC can retain β-catenin in the cytoplasm, leading to the inhibition of Wnt/β-catenin signaling. Silencing/overexpressing APC increased/decreased the total and phosphorylated β-catenin and the expressions of the Wnt downstream genes and stemness genes at both mRNA and protein levels (Fig. 4F to H and Fig. S15F to I). Further, softening/stiffened tumor cells facilitated/suppressed the formation and growth of tumor spheroids, while concurrently overexpressing/silencing APC diminished this effect and reduced/enhanced these abilities to the control or even lower/higher level (Fig. 4I to L). In addition, silencing/overexpressing APC enhanced/suppressed the colony formation and growth, which could be restored by inhibiting/activating Wnt signaling (Fig. 4M and N and Fig. S15AJ and K). These results suggest that actomyosin-mediated cellular mechanics regulate tumor cell self-renewal through cytoskeleton/APC/Wnt/β-catenin signaling.

### Cell mechanics dictate tumor cell self-renewal through Wnt-mediated Oct4 transcription

**Fig. 5.**
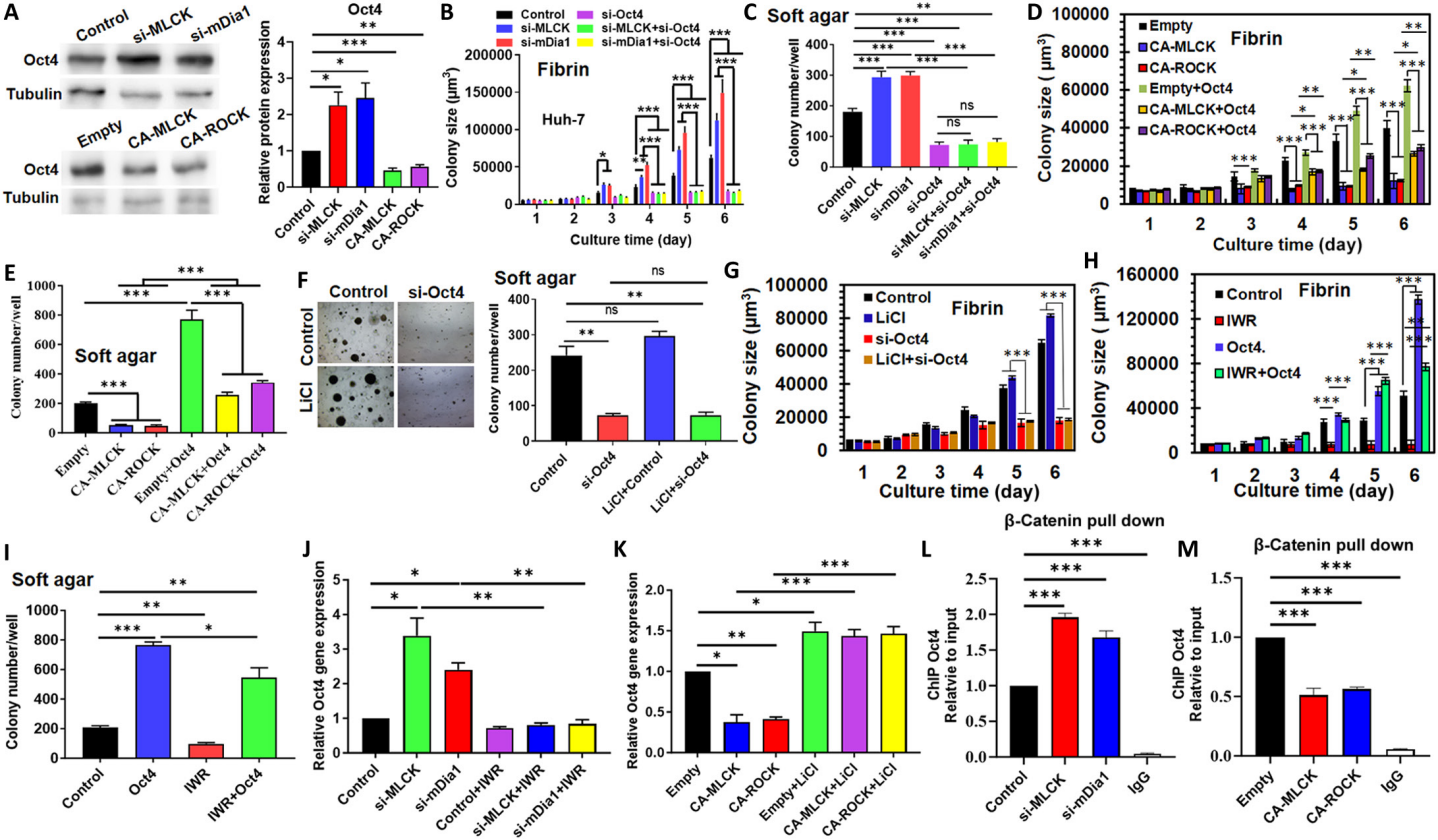
Cell mechanics-mediated Wnt signaling regulates self-renewal through β-catenin-induced Oct4 transcription. (A) Immunoblotting of Oct4 in softened and stiffened tumor cells. *n* = 3. (B and C) Formation and growth of tumor spheroids when softened Huh-7 cells were transfected with Oct4 siRNA. *n* = 3. (D and E) Formation and growth of tumor spheroids when stiffened Huh-7 cells were transfected with Oct4 plasmids. *n* = 3. (F and G) Formation and growth of tumor spheroids when Huh-7 cells were treated with LiCl and then transfected with Oct4 siRNA. *n* = 3. (H and I) Formation and growth of tumor spheroids when Huh-7 cells were treated with IWR and then transfected with Oct4 plasmids. *n* = 3. (J and K) mRNA expression of Oct4 analyzed by qRT-PCR in softened/stiffened Huh-7 cells that were treated with IWR/LiCl. *n* = 3. (L and M) Interaction between nuclear β-catenin and the promoter of Oct4 analyzed by the cleavage under targets and tagmentation (CUT&Tag) assay. *n* = 3. ANOVA was used for the statistical analysis together with the post hoc Bonferroni test. *P < 0.05; **P < 0.01; ***P < 0.001.

When Wnt/β-catenin signaling is activated, β-catenin translocates into the nucleus to initiate the transcription of target genes [34]. We aimed to further identify the downstream effector of cell mechanics-regulated Wnt/β-catenin signaling that sustains tumor cell self-renewal. Actomyosin-mediated cell mechanics influenced the transcription of multiple self-renewal genes (Figs. S4B and S9A and K), among which Oct4 appeared to be the most affected and could be one promising candidate due to its role in self-renewal [36]. We showed that softening cells up-regulated Oct4 protein by >120%, while stiffening cells down-regulated the expression by ~50% (Fig. 5A). Notably, silencing/overexpressing Oct4 diminished the promotive/suppressive effect of cell softening/stiffening on the formation and growth of tumor spheroids (Fig. 5B to E and Fig. S16E and F). These results suggest that cell mechanics regulate tumor cell self-renewal through Oct4.

Since APC affected both Wnt/β-catenin signaling and Oct4 expression (Fig. 4F to H), we next determined whether Oct4 functioned as a downstream effector to mediate the influence of APC–Wnt/β-catenin signaling on tumor cell self-renewal. Activating Wnt signaling moderately enhanced the growth but not the formation of tumor spheroids, while concurrently silencing Oct4 greatly inhibited these abilities (Fig. 5F and G). On the other hand, inhibiting Wnt signaling dramatically suppressed tumor cell self-renewal, while concurrently overexpressing Oct4 rescued this effect and enhanced both the formation and growth of tumor spheroids to the levels higher than that of control (Fig. 5H and I). At the transcriptional level, softening/stiffening cells up-/down-regulated Oct4 expression, which was reduced/enhanced to the control level after Wnt inhibition/activation (Fig. 5J and K). Further, the cleavage under targets and tagmentation (CUT&Tag) assay was conducted to analyze the interaction between nuclear β-catenin and the promoter of Oct4. Softening cells increased the binding of β-catenin with the promoter of Oct4 by 70 to 95%, while stiffening cells reduced this interaction by ~50% (Fig. 5L and M). These results collectively suggest that cell mechanics drive tumor cell self-renewal through APC/Wnt/β-catenin-mediated Oct4 transcription.

### Cytoskeleton/APC/β-catenin/Oct4 signaling is correlated with tumor differentiation and patient survival

**Fig. 6.**
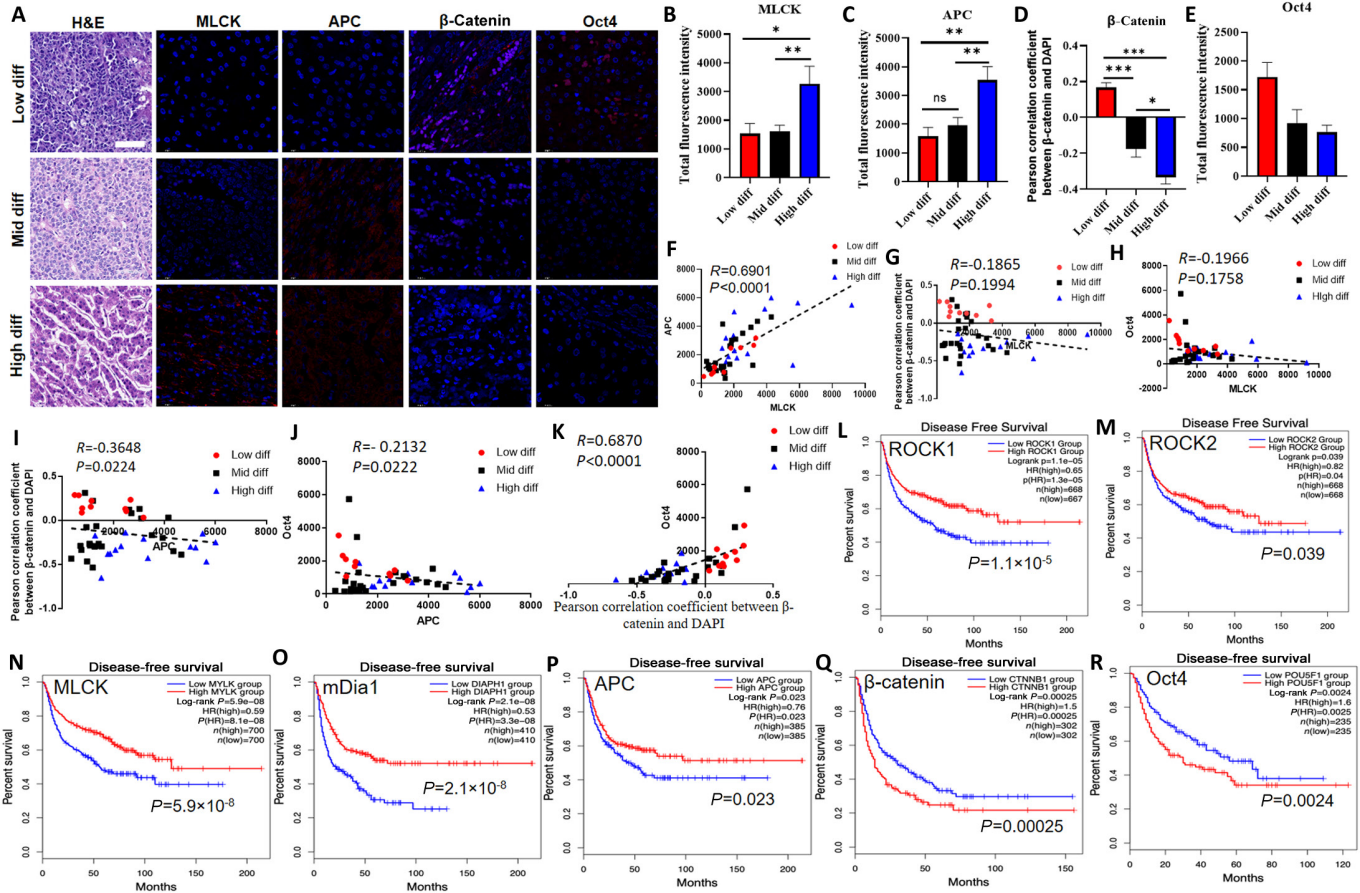
The cytoskeleton–APC–Wnt/β-catenin–Oct4 signaling is correlated with tumor differentiation and patient survival. (A to E) Expressions of MLCK, APC, and Oct4 and Pearson correlation coefficient between β-catenin and DAPI in clinical HCC samples with different differentiation levels analyzed by immunofluorescence staining. Patient samples were histologically graded into low, medium, and high differentiation level according to the Edmondson–Steiner grading system. The expressions of the indicated genes and the correlation between β-catenin and DAPI in (A) were quantified in (B) to (E) accordingly based on their differentiation state. Scale bar, 80 μm. *n* = 10, 25, and 14 for low, medium, and high differentiation level. ANOVA was used for the statistical analysis together with the post hoc Bonferroni test. (F to K) Correlation of the cytoskeleton–APC–Wnt/β-catenin–Oct4 signaling in clinical samples analyzed by linear regression. (L to R) Correlation between the expressions of the cytoskeleton–APC–Wnt/β-catenin–Oct4 signaling and patient survival. The Cancer Genome Atlas (TCGA) databases were utilized to analyze the relationship between the expressions of the indicated genes and patient survival. The statistics were conducted using multivariate Cox regression analysis.

We further explored the pathologic relevance of tumor cell mechanics and the regulatory signaling in clinical HCC samples. The activity of cytoskeleton/APC/β-catenin/Oct4 signaling was examined in patient-derived tumor tissues with low, median, and high differentiation states, which were histologically graded based on hematoxylin and eosin (H&E) staining and biopsy observation according to Edmondson–Steiner grade (Fig. 6A). The protein levels of MLCK and APC increased from low to high differentiation tumors (Fig. 6A to C), while nuclear localization of β-catenin decreased along with tumor differentiation (Fig. 6A and D). The Oct4 expression tended to reduce from low to high differentiation but without statistical significance (Fig. 6E). These results suggest that the activity of the regulatory signaling by which tumor cell mechanics drive self-renewal is associated with tumor differentiation. Further, we analyzed the relationship among these signaling proteins in patient samples. The protein expression of MLCK was correlated with APC positively and had a tendency to associate with the expressions of nuclear β-catenin and Oct4 negatively (Fig. 6F to H). The protein level of APC was negatively associated with nuclear localization of β-catenin and Oct4 expression (Fig. 6I and J), and nuclear β-catenin was positively correlated with Oct4 expression in tumor tissues (Fig. 6K). In addition, public databases were utilized to analyze the relationship between the mRNA expressions of cytoskeleton/APC/Wnt/β-catenin/Oct4 signaling and patient survival. The results showed that high expressions of cell mechanics-related genes (ROCK1, ROCK2, MLCK, and mDia1) and APC were associated with favorable patient survival (Fig. 6L to P), while the expressions of β-catenin and Oct4 were negatively correlated with the survival of HCC patients (Fig. 6Q and R). These findings suggest that the mechanism by which tumor cell mechanics regulate self-renewal and tumorigenicity is correlated in clinical tumor samples and associated with tumor differentiation and patient survival.

## Discussion

Tumor cells with high self-renewal and malignancy show reduced cellular stiffness [4,11–13], and soft tumor cells exhibit enhanced ability to self-renew [15], suggesting the close correlation between tumor cell mechanics and malignancy. Recent evidence implicates the suppressive effect of an actin regulator cancer-related regulator of actin dynamics (CRAD) on the tumorigenesis of colorectal cancer [37]. However, the roles of tumor cell cytoskeleton and the mechanics in tumor progression remain incompletely understood. This study demonstrates that the mechanical alteration of tumor cells during tumor progression is not trivial but drives self-renewal and tumorigenicity. In particular, low cellular stiffness, which may be mediated by genetic mutation, is necessary and sufficient in sustaining high malignancy (Fig. S16G). In line with this, recent studies show that elevating cortical tension or actomyosin contractility inhibits tumor cell migration and invasion [31,38–40]. Overexpressing F-actin regulator KIAA1211 increases tumor cell stiffness but suppresses lung metastasis [41]. Activating actin polymerization enhances the cytotoxic T cell-induced killing [17]. Reinforcing the membrane-to-cortex attachment and membrane tension blunts the metastasis of invasive cancer cells [42]. The low cellular stiffness enhances cellular uptake of drug-loaded nanoparticles or tumor cell-derived microparticles, which mediates effective eradication of soft and malignant tumor cells [13,14]. Therefore, this study has unveiled the regulatory role of intrinsic cell mechanics in tumorigenicity and identified tumor cell mechanics as promising targets for the development of mechanotargeting strategies for cancer therapy. Compared to various biochemical cues that usually function in one specific cancer type or even subtype due to intratumoral and intertumoral heterogeneity, the alterations in tumor cell mechanics are more generalized and regulate self-renewal in multiple types of cancer, which may bestow advantages to the therapeutics that target tumor cell mechanics.

Cell cytoskeleton is intertwined with many intracellular organelles and signaling molecules [43]. Targeting actin cytoskeleton modulates not only cell mechanics but also likely other cellular processes. It is thus possible that altering actomyosin may regulate tumor cell malignancy independent of the effect on cell mechanics. To alleviate this concern, this study targets multiple molecules in 2 different pathways, namely, actin polymerization (e.g., F-actin, mDia1, and α-actinin) and Rho/ROCK/myosin signaling (e.g., RhoA, MLCK, ROCK, and myosin II), which have similar effects on self-renewal but no obvious influence on cell morphology, survival, and proliferation. The regulation of self-renewal by cell mechanics is reversible. Further, the effect of cytoskeletal alteration on self-renewal can be rescued by targeting myosin activity, while the influence of myosin can be diminished by modulating cytoskeleton. All these suggest that the influence of actomyosin activity on self-renewal and tumorigenicity is probably through the effect on cell mechanics. Nevertheless, the attempt to clarify this issue is challenged by the lack of a direct target that is only/closely related to cell mechanics.

Actomyosin-mediated cell mechanics regulate tumor cell self-renewal through cytoskeleton/APC/Wnt/β-catenin-Oct4 signaling (Fig. S16G), which is relevant in the clinical samples and associated with tumor differentiation and patient survival. Note that ~27% of HCC cases (e.g., HepG2) contain mutations in *CTNNB1* gene (encoding β-catenin), leading to aberrant activation of the Wnt/β-catenin pathway that is no longer affected by the protein destruction complex [44]. Nevertheless, HCC cells (e.g., Huh-7, PLC/PRF/5, and SNU387) that do not harbor such mutations can respond to the stimulation signals of Wnt [45], including cytoskeleton-mediated β-catenin association with and dissociation from APC. In particular, weakening/strengthening actin cytoskeleton reduces/enhances the binding of β-catenin with APC and further facilitates the nuclear/cytoplasmic localization. Nuclear β-catenin interacts with the promoter of Oct4 and enhances the gene transcription possibly in cooperation with T cell factor and lymphoid enhancer-binding protein transcription factor. The stable interaction of β-catenin with APC leads to its cytoplasmic retention and degradation, which inactivates Wnt/β-catenin signaling. Further, softening/stiffening cells decreases/increases the colocalization between F-actin and APC, which may presumably weaken/strengthen the interaction between APC and β-catenin. APC can interact with actin and microtubule via its basic domain [33] and can also bind to α-catenin [35], a linker between actin cytoskeleton and the cell membrane, both of which may affect the interaction between APC and β-catenin. Despite the effect on the stability of β-catenin, APC also interacts with CtBP in the nucleus to repress the transcription of Wnt target genes [35,46]. Our results are in line with the recent findings that loss of CRAD in colorectal cancer disrupts F-actin polymerization and cadherin–catenin–actin complex, which then activates Wnt/β-catenin signaling and promotes tumorigenesis [37]. Soft microenvironments down-regulate CRAD, retain YAP in the cytoplasm, and up-regulate stemness genes Oct4 and Nanog, which facilitate stemness and metastasis [41].

Tumor progression is associated with not only cytoskeletal remodeling and tumor cell softening but also tumor tissue stiffening. The overall tumor tissues are generally stiffer than their healthy counterparts in multiple types of cancer mainly due to excessive deposition of collagen and matrix crosslinking [47,48], which has been proposed as one important biophysical trait of cancer [49]. It is believed that tumor and stromal cells perceive the stiffened tumor tissue mainly through integrin-mediated mechanotransduction, which influences a variety of malignant functions, such as tumor growth [48,50], invasion [51], intravasation [52], chemoresistance and dormancy [53], and metastatic colonization [54]. Recent evidence shows that stiff matrices enhance the stemness of liver and colorectal cancer stem cells through the activation of YAP signaling [55,56]. Further, a latest work has made the efforts to address this paradox of soft cells and stiff niches within tumor tissues [57]. However, it remains unclear how these two mechanical phenomena evolve during tumor progression and how they separately or synergistically influence tumor malignancy.

## Conclusion

This study reports that moderately softening tumor cells promotes the self-renewal and tumorigenicity, while stiffening cells inhibits these abilities, which can be applicable to multiple types of cancer. Mechanistically, softening/stiffening actin cytoskeleton weakens/strengthens the interaction between APC and β-catenin, which leads to β-catenin nuclear/cytoplasmic localization. The cytoplasmic retention results in protein degradation and eventually inactivates Wnt/β-catenin signaling. Nuclear β-catenin binds to the promoter of Oct4, which facilitates the gene transcription that is crucial in sustaining tumor cell self-renewal. Further, cell mechanics regulate the interconversion between CSCs and non-CSCs, which depends on Wnt signaling. In summary, this study demonstrates that actomyosin-mediated cell mechanics drive tumor cell self-renewal and tumorigenicity via cytoskeleton/APC/Wnt/β-catenin/Oct4 signaling, which are clinically relevant and associated with patient survival. Therefore, our research unveils cell mechanics as a driving force for tumor malignancy and opens a new avenue of mechanomedicine strategies for cancer therapy that target the mechanics of tumor cell cytoskeleton.

## Methods

### Cell lines and cell culture

Human HCC cell lines Huh-7 (Japanese Cancer Research Resources Bank, catalog no. JCRB0403), PLC/PRF/5 [American Type Culture Collection (ATCC), catalog no. CRL-8024], HepG2 (ATCC, catalog no. HB-8065), Hep3B (ATCC, catalog no. HB-8064), and MHCC97-L (Beyotime, catalog no. C6586) (gifts from C. L. C. Wong in the University of Hong Kong), human breast cancer cell line MCF-7 (ATCC, catalog no. HTB-22), cervical cancer cell line HeLa (ATCC, catalog no. CRM-CCL-2), colon cancer cell line CT26 (ATCC, catalog no. CRL-2638), and non-small cell lung cancer cell line A549 (ATCC, catalog no. CRM-CCL-185) were cultured in Dulbecco's Modified Eagle Medium (DMEM; HyClone, Logan, UT) with 10% fetal bovine serum (FBS; HyClone) and 1% penicillin/streptomycin (HyClone). All cells were authenticated and maintained in a cell culture incubator under a 95% air and 5% CO_2_ humidified environment at 37 °C and passaged every 3 to 4 days with 0.25% trypsin (HyClone). The cells used for the experiments involved in this study were no more than 20 passages.

### Pharmacologic treatment

To soften tumor cells, HCC cells were pretreated with different doses of Y-27632 (2 and 20 μM; Selleckchem), blebbistatin (6 and 50 μM; Selleckchem), and cytochalasin D (0.1 and 1 μM; Selleckchem) for 48 h. To stiffen them, the cells were pretreated with 20 nM jasplakinolide (Tocris, #2792/100U) or 1 nM narciclasine (MedChemExpress) for 12 h. Nocodazole (1 and 6 μM; Abcam) and withaferin A (WFA; Abcam; 1, 2, and 5 μM) were used to disrupt microtubules and vimentin, respectively. The Wnt activator LiCl (2 μM) and inhibitor IWR-1-endo (0.2 μM) were used to activate and inhibit the Wnt/β-catenin pathway, respectively.

### Cell transfection with plasmids

CA forms of MLCK and ROCK mutants were lentiviral vector pSLIK containing the TRE tight doxycycline-inducible promoter and YFP variant Venus (RRID: Addgene_84647; Addgene_84649) and were gifts from S. Kumar (31). Viral particles were packaged in 293T cells and used to infect cancer cells at a multiplicity of infection of 1 IU/cell. In brief, 293T cells were seeded to be 70 to 90% confluent in a 6-well plate and allowed to settle down overnight. For each well, 7.5 μl of Lipofectamine 3000 reagent (Thermo Fisher Scientific) was diluted in 125 μl of Opti-MEM medium and mixed well. The master mix of 2,500 ng of DNA was diluted by 125 μl of Opti-MEM, and 5 μl of P3000 reagent was then added and mixed well. Next, 125 μl of diluted Lipofectamine 3000 reagent was added to each tube of DNA master mix with the ratio of 1:1. The total mixture was incubated at room temperature for 10 to 15 min. To genetically stiffen cells, this mixture was added to 293T cells. The supernatant medium containing the well-packaged viral of CA-MLCK and CA-ROCK was used to infect target cells. After 48 h, the transfection medium was removed and doxycycline (50 ng/ml) (TargetMol) was added to activate the CA plasmids for at least 2 days.

pCMV-Neo-Bam APC was a gift from B. Vogelstein (RRID: Addgene_16507) [58]. Tumor cells were seeded in 6-well plates and transfected with 2 μg of pCMV-Neo-Bam APC or empty vectors using Lipofectamine 3000 reagent for 48 h before further experiments.

### RNA interference

Short interfering RNAs (siRNAs) were used to inhibit the transcription of target genes. Cells were seeded to be 40 to 60% confluent in a 6-well plate and allowed to settle down overnight. For each well, 7.5 μl of Lipofectamine 3000 reagent (Thermo Fisher Scientific) was diluted in 250 μl of Opti-MEM medium and mixed well. Then, 1 μl of siRNA (20 μM stock) was diluted in 250 μl of Opti-MEM. Next, 250 μl of diluted Lipofectamine 3000 reagent was added to each tube of siRNA mix with the ratio of 1:1. The total mix (500 μl) was incubated at room temperature for 10 to 15 min and then added to target cells in 1.5 ml of full medium. The transfected cells were visualized and used for experiments after 24 to 72 h of transfection.

### Cell proliferation and viability assay

Cells were seeded in 96-well plates at the density of 2 × 10^3^ and 5 × 10^3^ cells per well and settled down overnight for the proliferation and viability assay, respectively. At the indicated time points, the number of viable cells was measured by MTS assay. In brief, the medium was removed and 20 μl of sterilized CellTiter 96 Aqueous One Solution (5 mg/ml, Promega) was added to each well. After 4 h of incubation in darkness at 37 °C, the absorbance of each well was measured at 490 nm by the Benchmark Plus microplate reader (Bio-Rad). The experiment was carried out 3 times with at least 3 wells per condition each time.

### Cell stiffness measurement by atomic force microscopy

An atomic force microscope (AFM; Bruker Catalyst) combined with an inverted optical microscope (Nikon) was used to measure cell stiffness. The silicon nitride cantilevers with a spring constant *k* of 0.02 to 0.08 N/m and tip radius of 20 nm were chosen to probe the cells in a 60-mm dish for the experiment at room temperature. The scan size for all measurements was set to 0 to maintain a constant position over a cell, and the tip was brought into contact with the cell at the indicated regions. The force–indentation curves were recorded at 1 Hz to estimate the cell stiffness. For each cell, the force–indentation curves were obtained from at least 5 different cellular locations and the averaged value was used to represent the stiffness for this cell. The force *F* between the tip and the cell was the product of the cantilever deflection δ and *k*, i.e., *F* = *k* × δ. The Young’s modulus *E* could be calculated by fitting the force–indentation curves with Hertzian model for a pyramidal tip, i.e., *F* = 2/π × tan(α) × *E*/(1 − *v*^2^) × *d*^2^, where α is the half tip angle, the Poisson's ratio *v* is set to 0.5, and *d* is the indentation depth. *d* was kept within 500 nm at 1 Hz to avoid potential substrate effects and cell damage.

### Traction force microscopy

Briefly, fluorescent beads (200 nm in diameter) were embedded into the surface of 5-kPa polyacrylamide gels (12). When living cells were cultured on the gels for 12 h, the images of the beads under a cell were captured before and after removing the cell, and further utilized to calculate the bead displacement, from which the traction field could be computed using the inverse Boussinesq mathematical model.

### Fibrin gel preparation

After trypsin treatment, cells were suspended in DMEM with a density at 10^5^ cells/ml. Fibrinogen was diluted into 2 mg/ml concentration with T7 buffer (50 mM tris, 150 mM NaCl, pH 7.4) and mixed with the same volume of cell solution, resulting in 1 mg/ml fibrinogen. The mixture (50 μl) was seeded into one well of a 96-well plate, which had been preadded with 5 μl of thrombin (4 U/ml). The solution was gently mixed by pipetting up and down twice and swirling to spread the gel throughout the whole well. The plate was transferred to a 37 °C cell culture incubator and allow the gel solution to solidify for 10 min. Finally, cell culture medium was added into each well. To measure the size of the generated tumor spheroids, at least 30 colonies were captured randomly every day. ImageJ [National Institutes of Health (NIH)] was used to analyze the colony size.

To collect fibrin-selected cells, the gels were dissolved using a solution mixture of collagenase (Sigma, C0130) and dispase II (Sigma, D4693) at the final concentration of 0.08% and 0.4% (w/v), respectively. The solution (400 μl) was added to each well (24-well plate) containing 300 μl of fibrin gel with 600 μl of existing culture medium for 30 min at 37 °C. After the gel was dissolved, the colonies were pipetted gently into single cells. The cells were then washed twice by phosphate-buffered saline (PBS).

### Colony formation assay in soft agar

The soft agar method can evaluate cell stemness and carcinogenesis based on anchorage-independent cell growth. In brief, 2 ml of 1% agarose (Sigma) was added onto each well in a 6-well plate and allowed to cool down for 30 min at room temperature to form the bottom layer. Cell suspensions (4 × 10^4^ cells/ml) were then mixed with 0.8% agarose at 1:1 ratio to form the agar gel in the 6-well plate, which was kept at 4 °C for 10 min and then incubated at 37 °C with 5% CO_2_. Four to five drops of complete cell culture medium were added to the well every 4 days. After 28-day incubation, the colonies were stained with 0.5 ml of 0.005% crystal violet (Thermo Fisher Scientific) for 1 h in darkness and counted under the microscope. For each condition, at least 3 wells of soft agar were prepared and analyzed.

### Sphere formation assay

Cells were suspended in PromoCell 3D Tumorsphere Medium XF (PromoCell) at the concentration of 10,000 cells/ml in a 6-well suspension culture plate (1ml per well). Fresh PromoCell 3D Tumorsphere Medium XF (0.5 ml) was added every 3 to 4 days. The colony number was counted at day 7. For each condition, at least 3 wells were prepared and analyzed.

### Quantitative real-time PCR

Total mRNAs were extracted by the Aurum Total RNA Mini Kit (Bio-Rad), and complementary DNA was synthesized using the RevertAid First Strand cDNA Synthesis Kit (Thermo) according to the manufacturer’s instructions. Quantitative real-time polymerase chain reaction (qRT-PCR) was performed using Forget-Me-Not EvaGreen qPCR Master Mix with Rox (Biotium) and CFX96 Real-Time System (Bio-Rad). The sequences of all the primers were obtained from the National Center for Biotechnology Information (NCBI) database and listed in Table S1. For data analysis, the expressions of all genes were normalized using the ΔΔcycle threshold method against human glyceraldehyde-3-phosphate dehydrogenase (GAPDH).

### Flow cytometry analysis

Flow cytometry analysis was performed using the BD Accuri C6 Flow Cytometer (Accuri Cytometers Inc.) and CFlow software (Accuri Cytometers Inc.). Cells were detached by 0.2% EDTA in PBS and incubated at 37 °C for 30 min. Cells were washed twice by PBS and centrifuged at 1,000 rpm for 5 min. DMEM with 2% FBS was used to dilute antibodies and incubate cells at 4 °C for 30 min. For each experiment, an unstained group was prepared in order to exclude the autofluorescence. Cells were then rinsed with PBS and resuspended in 200 μl of 2% FBS in PBS. The cells were filtered through a 60-μm cell strainer to obtain single-cell suspension before sorting. The percentage of the indicated subpopulation was then detected by a BD Accuri C6 flow cytometer.

### Fluorescence-activated cell sorting

Cells were pretreated as described in flow cytometry analysis but using the BD FACSAria III Cell Sorter for sorting (BD Biosciences). Cells were detached by 0.2% EDTA, washed twice using PBS, and then cocultured with the EpCAM antibody (Abcam, catalog no. ab223582; 10^6^ cells/ml). The antibody was diluted to 1:2,000 using 2% FBS in PBS, and the cells were cocultured with the diluted antibody on ice for 30 min. After the centrifugation and washing, cells were sorted to EpCAM^+^ and EpCAM^−^ cells by the BD FACSAria Cell Sorter. Unsorted cells were set as control. Sorted cells were centrifuged, resuspended in full medium, and cultured in petri dishes for further experiments.

### F-actin staining

Cells were plated on gelatin-coated coverslips in a 24-well plate overnight and then fixed with 4% formaldehyde (Sigma-Aldrich) for 30 min at room temperature. The fixed coverslips were washed 3 times with PBS and then incubated with 0.1% Triton X-100 (SAFC) in 1% bovine serum albumin (BSA) for 1 h at room temperature for permeabilization. After washing the cells with PBS, 1× green fluorescent phalloidin conjugate working solution (Abcam) was added to the cells for 1 h. After gentle washing, the cells were counterstained with DAPI (4′,6-diamidino-2-phenylindole) (Thermo Fisher Scientific) for nuclear staining. For each condition, at least 100 cells were imaged using the inverted fluorescent microscope (Nikon). The fluorescence intensity was analyzed by ImageJ (NIH).

### Immunofluorescence staining

Cells were treated similarly as described in F-actin staining. To stain the specific targets, the cells were incubated with the corresponding primary antibodies in 1% BSA overnight at 4 °C. All antibodies were from Abcam with the dilution ratios listed below: α-tubulin (Abcam, catalog no. ab7291; 1:1,000), vinculin (Abcam, catalog no. ab219649; 1:1,000), vimentin (Abcam, catalog no. ab92547; 1:1,000), APC (Abcam, catalog no. ab16794; 1:200), β-catenin (Abcam, catalog no. ab32572; 1:250), Oct4 (Abcam, catalog no. ab181557; 1:1,000), and CD133 (Abcam, catalog no. ab19898; 1:1,000). The coverslips were then washed with PBS and incubated with goat anti-rabbit immunoglobulin G (IgG) H&L (Alexa Fluor 488) (Abcam, catalog no. ab6702), goat anti-mouse IgG (H+L) highly cross-adsorbed secondary antibody, and Alexa Fluor Plus 488 (Invitrogen) in 1% BSA for 1 h at room temperature, respectively. After gentle washing, the cells were counterstained with DAPI (Thermo Fisher Scientific, catalog no. D1306) for nuclear staining. For each condition, at least 100 cells were imaged using the inverted fluorescent microscope (Nikon). The fluorescence intensity was analyzed by ImageJ.

### Western blot

Proteins were extracted from the lysed cells with a Mem-PER Plus membrane protein extraction kit (Thermo Fisher Scientific, catalog no. 89842). Trans-Blot Turbo (Bio-Rad) was used to transfer proteins from 8% sodium dodecyl sulfate–polyacrylamide gel electrophoresis (SDS-PAGE) gel to a polyvinylidene difluoride Western blot membrane. The transferred membrane was incubated with 3% BSA and then with the primary antibodies at 4 °C overnight, including phospho-β-catenin-Ser^675^ (ABclonal, catalog no. AP0795), phospho-myosin light chain 2 (Ser^19^) (Cell Signaling, catalog no.3671), β-catenin (ABclonal, catalog no. A0316), tubulin (Abcam, catalog no. ab7291), APC (Abcam, catalog no. ab40778), Oct4 (Abcam, catalog no. ab181557), and Bmi1 (Abcam, catalog no. ab126783). The membrane was washed and incubated with the secondary antibodies, goat anti-mouse IgG (H+L)-HRP (horseradish peroxidase) conjugate and goat anti-rabbit IgG (H+L)-HRP conjugate (Bio-Rad, catalog no. 1706516), for 2 h. GAPDH (Abcam) was used for reference. The membranes were then incubated with Clarity and Clarity Max Western ECL Blotting Substrates (Thermo Fisher Scientific). Images were captured using the ChemiDoc Imaging system (Thermo Fisher Scientific).

### Coimmunoprecipitation assay

Cells were detached by trypsin and washed twice by PBS. Proteins were extracted from the lysed cells by Mem-PER Plus membrane protein extraction kit (Thermo Fisher Scientific, catalog no. 89842). The extracted protein was then precleared with protein A agarose beads (SMART Lifescience). The APC antibody (Abcam, catalog no. ab40778) was added with protein A agarose beads for 30 min and then coincubated with cleared protein for 1 h at room temperature. Appropriate IgG was used as a negative control. Immunocomplexes were washed 5 times and then boiled in SDS sample buffer for 5 min. Proteins were analyzed using Western blots as described above.

### Animal experiment

Six-week-old BALB nude mice were separated by sex and randomly grouped. To develop the subcutaneous xenograft model, 2 × 10^6^ of softened (Control, si-MLCK, si-mDia1) or stiffened (Empty, CA-MLCK, CA-ROCK) Huh-7 cells were mixed with the same volume of Matrigel solution (Corning Matrigel Matrix Phenol Red-free, #356237) and then subcutaneously injected into the right hind flanks of mice. For the rescue experiment, IWR (25 mmol/kg/day) was intraperitoneally injected into the mice with the softened cells, while LiCl (10 mmol/kg/day) was used to treat the mice with the stiffened cells. The tumor size was measured every 4 days, and the tumor volume was calculated as volume = 1/2 × width × length^2^. All mice were euthanized at the end of the experiment, and tumor tissues were retrieved and weighed.

To develop the orthotopic xenograft model, the mice were anesthetized by ketamine–xylazine–PBS (1.0 ml of ketamine + 0.5 ml of xylazine + 8.5 ml of PBS, 10 μl/g). The skin was disinfected using 75% ethanol for 3 times and gripped with forceps to make an approximately 1 cm horizontal incision from the midline toward the left upper abdomen. A cotton-tipped applicator was inserted into the left upper abdomen under the left lobe of the liver and then removed slowly. For each mouse, 10^6^ of softened, stiffened, or control MHCC97L cells were mixed with the same volume of Matrigel solution (20 μl mix each mice). After the injection, a gentle pressure was applied on the needle insertion site with a cotton-tipped applicator for several minutes to stop bleeding. Bupivacaine was used to release the pain and injury caused by the surgery process every 12 h up to 48 h. The luminescence signal was detected every week.

### Limiting dilution assay

Different numbers of cells were injected subcutaneously into 6-week-old nude mice following the same protocol as described above. The tumor incidence was examined at week 8. Mice with no sign of tumor formation were sacrificed 2 months after injection, and the injection sites were carefully examined to confirm no tumor development via surgical incision and exploration. The stem cell frequency in each condition was calculated by the extreme limiting dilution analysis via http://bioinf.wehi.edu.au/software/elda/.

### CUT&Tag assay

To detect the binding of β-catenin protein with the DNA sequence of Oct4 in softened and stiffened tumor cells, the NovoNGS CUT&Tag 2.0 High-Sensitivity Kit (NovoProtein, N259-YH01) was used to conduct the CUT&Tag assay. Appropriate IgG was used as a negative control. Cells (5.0 × 10^5^) were washed twice with 1.5 ml of wash buffer and then mixed with activated concanavalin A beads. After successive incubations with the primary antibody of β-catenin (ab32572, Abcam) at room temperature for 2 h and the secondary antibody (Abcam) at room temperature for 1 h, the cells were washed and incubated with pAG-Tn5 for 1 h. Then, MgCl_2_ was added to activate tagmentation for 1 h. The tagmentation reaction was then stopped, and the expression of Oct4 in the transposed DNA fragments was detected by qPCR.

### β-Catenin reporter assay

The β-catenin reporter plasmid Super 8x TOPFlash (Super-TopFlash) and its mutant control Super 8x FOPFlash (FopFlash) were provided by T. K. W. Lee from the Hong Kong Polytechnic University. Cells (1 × 10^4^) were seeded in a 96-well plate overnight before transfection. For knockdown groups, the cells were cotransfected with 50 ng of TopFlash or FopFlash plasmids together with the corresponding siRNAs using Lipofectamine 3000. For constitutive activation groups, cells were transfected with 50 ng of TopFlash or FopFlash plasmids after lentivirus transfection and treated with doxycycline. The bioluminescent signal driven by β-catenin activity was measured at 48 h after transfection using Firefly Luc One-Step Glow Assay Kit (Thermo Fisher Scientific) according to the instructions. The Top/Fop ratio was used to index the transcription activity of β-catenin.

### Histopathology and immunofluorescence of patient samples

Tumor tissues from patients with HCC were paraffin-embedded and sectioned with equal thickness from the Department of Pathology, Guizhou Provincial People's Hospital. Ethical approval was granted by the Institutional Human Ethics Committee, Hong Kong Polytechnic University, Chongqing University, and Guizhou Provincial People's Hospital. Paraffin tumor sections were stained with H&E and imaged using the Pannoramic MIDI Scanner (3DHISTECH, HU). According to the results of H&E staining and biopsy observation, the sample sections were graded according to Edmondson–Steiner grade. A representative snapshot of each grade was obtained by CaseViewer software.

Immunofluorescence staining of the paraffin sections was conducted following a protocol reported previously [59]. In brief, after a series of staining preparation of paraffin sections, the samples were incubated with the primary antibodies overnight at 4 °C, then with the secondary antibodies for 1 h at 37 °C, and finally with the DAPI-containing mounting medium (Solarbio, catalog no. S2110). The following primary antibodies were used: mouse anti-Oct4 at 1:200 (Zenbio, catalog no. 220487), mouse anti-β-catenin at 1:200 (Proteintech, catalog no. 66379-1-Ig), rabbit anti-APC at 1:200 (ABclonal, catalog no. A2818), and rabbit anti-MYLK at 1:200 (Proteintech, catalog no. 21642-1-AP). Goat anti-rabbit IgG conjugated with Alexa Fluor 488 (1:500, Abcam, catalog no. ab150077) and goat anti-mouse IgG conjugated with Alexa Fluor 568 (1:500, Abcam, catalog no. ab175473) were used as fluorescently labeled secondary antibodies. All the images were visualized and captured under a laser-scanning confocal microscope (Leica SP8) with the same image acquisition parameters. The fluorescence intensity was calculated using ImageJ software (NIH).

### The Cancer Genome Atlas data

HCC data were retrieved from The Cancer Genome Atlas (TCGA: http://cancergenome.nih.gov/; RRID:SCR_003193) using the TCGA data portal to download the clinical data and Infinium. Overall survival of high/low expression of mDia1, APC, β-catenin, and Oct4 genes was analyzed in the TCGA KIRC project. The group cutoff was set as median, cutoff-high 50%, and cutoff-low 50%. The hazard ratios were calculated based on Cox PH Model.

### Statistical analysis

All the results were represented by mean ± SEM. The statistics between 2 conditions and among 3 or more conditions were analyzed by 2-tailed Student’s *t* test and analysis of variance (ANOVA), respectively. The post hoc Tukey or Bonferroni test was adopted in the ANOVA analysis for the comparisons with equal or unequal sample sizes. *P < 0.05; **P < 0.01; ***P < 0.001. The stem cell frequency and *P* value were calculated using the tool at http://bioinf.wehi.edu.au/software/elda/.

## Data Availability

The data are available from the authors upon reasonable requests.
