# Supplementary material for: The Mechanics of Tumor Cells Dictate Malignancy via Cytoskeleton-Mediated APC/Wnt/β-Catenin Signaling
Source: Research (Wash D C). 2023 Sep 21;6:0224. doi: 10.34133/research.0224 (PMC10513157; doi:10.34133/research.0224)
Supplement: Supplementary 1 — Table S1 Figs. S1 to S16 [file research.0224.f1.pdf]

The mechanics of tumor cells dictate malignancy via cytoskeleton-mediated APC/Wnt/β-catenin signaling

Xi Chen[1,3,4#], Zichen Xu[2,4#], Kai Tang[4#], Guanshuo Hu[1,3,4], Pengyu Du[4], Junfan Wang[4], Cunyu Zhang[3,4], Ying Xin[4], Keming Li[4], Qianting Zhang[2], Jianjun Hu[5], Zhuxue Zhang[5], Mo Yang[4], Guixue Wang[2*], Youhua Tan[1,3,4*]

Table S1 Primers for reverse transcription PCR (RT-PCR)

	**5' primer**	**3' primer**
**ACTN1**	TGAGGAGTGGTTGCTGAATGAG	AACTTCTCTGCCAGGTGGTCC
**APC**	GCAGATTCTGCTAATACCCT	AACAGCTTTGTGCCTGGCT
**β-catenin**	TTGATGGAGTTGGACATGGC	AGTGAAGGAATGAAGAAAATCC
**Bmi1**	TTCATTGATGCCACAACCAT	CAGCATCAGCAGAAGGATGA
**CD133**	CAGCATCAGCAGAAGGATGA	ATCCATTCCCTGTGCGTTGA
**c-Myc**	GGCTCCTGGCAAAAGGTC	CTGCGTAGTTGTGCTGATGT
**CTNNA1**	CAGCTAGCCGCAGAAATGAC	GAGGCTCCAACAGTCTCTCAA
**EpCAM**	GCAGCTCAGGAAGAATGTG	CAGCCAGCTTTGAGCAAATGAC
**GAPDH**	GCGACACCCACTCCTCCACCTTT	TGCTGTAGCCAAATTCGTTGTCATA
**MLCK**	CACCGTCCATGAAAAGAAGAGTAG	GAGAGGCCCTGCAGGAAGATGG
**Nanog**	TTTGTGG- GCCTGAAGAAAACT	AGGGCTGTCCTGAA TAAGCAG
**Oct4**	CTGGGTTGATCCTCGGACCT	CCATCGGAGTTGCTCTCCA
**Sox2**	GCCGAGTGGAAAC- TTTTGTCG	GGCAGCGTGTACTTATCCTTCT

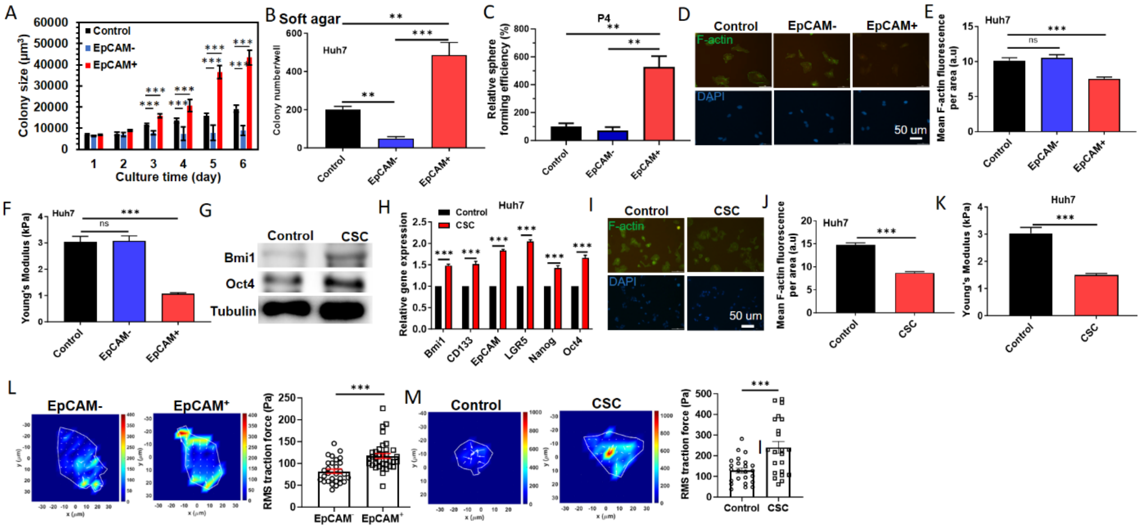

Figure S1 Tumor cell mechanics are correlated with self-renewal in HCC. EpCAM+ HCC cells generate larger tumor spheroids in soft fibrin (A) and more colonies in soft agar (B) and sphere formation assay at passage 4 (C) compared to EpCAM- cells. Fluorescence-activated cell sorting (FACS) was utilized to sort EpCAM+/- subpopulations from Huh-7 cells. The self-renewal of the sorted tumor cells was tested in soft fibrin and soft agar. n=3. EpCAM+ cells exhibit lower F-actin (D, E) and cellular stiffness (F) than EpCAM- cells. F-actin was examined by phalloidin staining and cell stiffness was measured by atomic force microscopy. n>60 cells in (E) and n>90 cells in (F). (G, H) Fibrin-selected CSCs up-regulate self-renewal genes. HCC CSCs were obtained by culturing Huh-7 cells in soft fibrin gels for 7 days. The expressions of the indicated self-renewal genes were measured by both qRT-PCR (H) and western blotting (G). The results in (G) were representative of two independent experiments. n=3 in (H). Fibrin-selected CSCs show lower F-actin (I, J) and cellular stiffness (K) than control cells. n>70 cells in (J). n>110 cells in (K). (L, M) EpCAM+ cells and fibrin-selected CSCs generate higher contractility than EpCAM+ cells and control cells. Cellular contractility was represented by traction force measured by traction force microscopy. n>24 cells for each condition. Scale bar in (D and I): 50 μm. Mean ± SEM. One-way ANOVA and student t-test were used for the

statistical analysis in (A, B, E, and F) and (H and J-M), respectively. *, p<0.05; **, p<0.01; ***, p<0.001. ns: no significance.

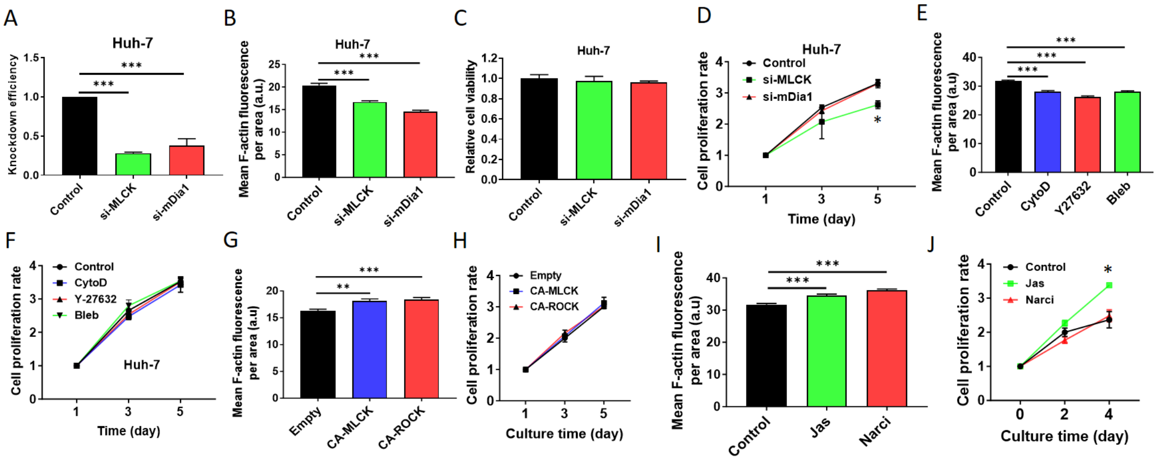

Figure S2 The effect of cell mechanics on cytoskeleton and proliferation. (A) The knockdown efficiency of MLCK and mDia1 siRNAs. n=3. (B) F-actin level after the knockdown of MLCK and mDia1. n>100 cells. Tumor cell viability (C) and proliferation (D) after the knockdown of MLCK and mDia1. n=3. F-actin level (E) and proliferation (F) of tumor cells after the treatment of Cytochalasin D (CytoD), Y27632, and Blebbistatin (Bleb). n>100 cells in (E); n=3 in (F). F-actin level (G) and proliferation (H) of tumor cells transfected with CA-MLCK and CA-ROCK plasmids. n>100 cells in (G); n=3 in (H). F-actin (I) and proliferation profile (J) of tumor cells after the treatment of Jas and Narci. n>100 cells in (I); n=3 in (J). One-way ANOVA was used for the statistical analysis. *, p<0.05; **, p<0.01; ***, p<0.001.

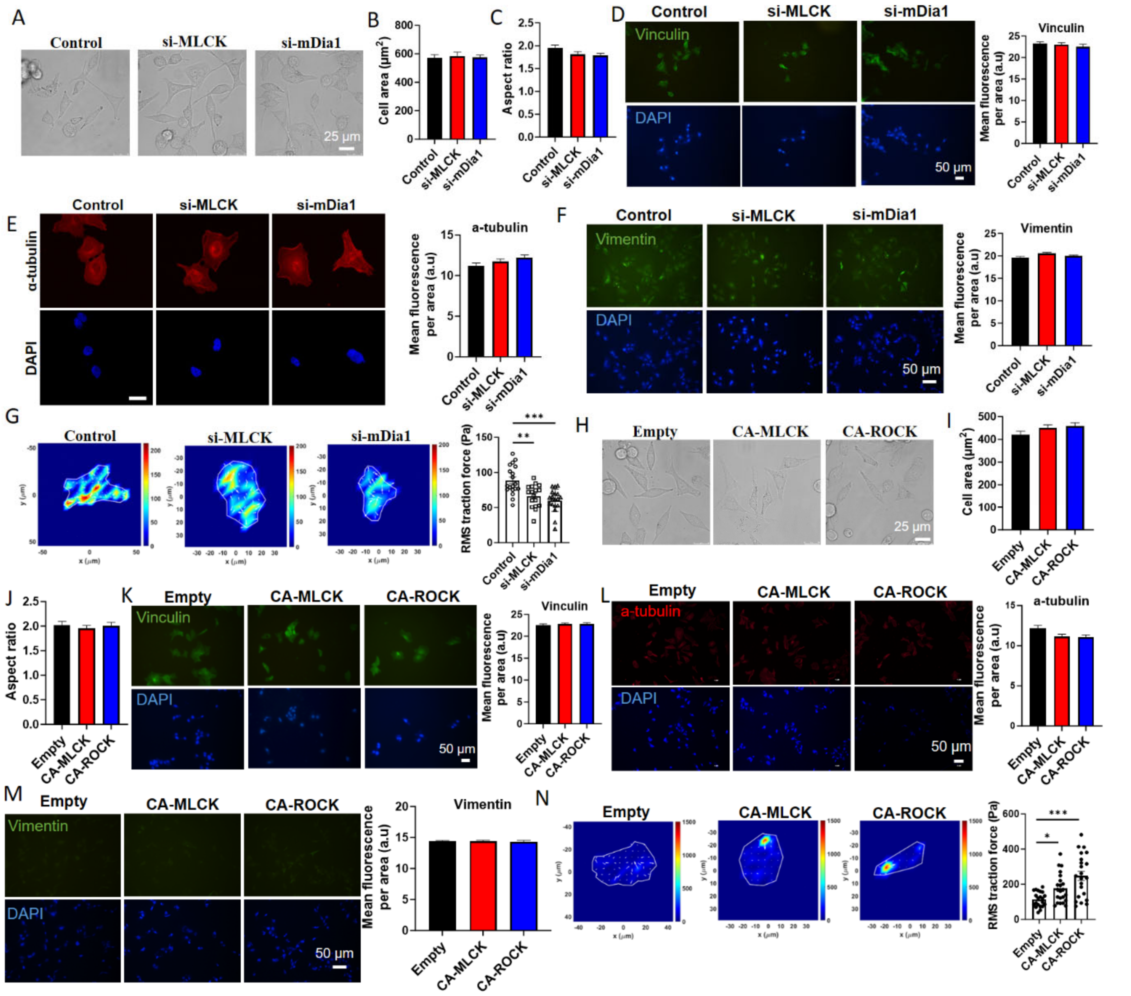

Figure S3 Modulating cell mechanics has no significant influence on cell morphology, focal adhesion, microtubule, and intermediate filament while affecting cellular contractility. (A-C) Softening cells has no effect on cell morphology. Huh-7 cells were transfected with the siRNAs of MLCK and mDia1. The spreading area and aspect ratio of these cells were measured. n>100 cells in (B, C). (D-F) Softening cells has no effect on focal adhesion, microtubule, and intermediate filament. The expressions of vinculin, microtubule, and vimentin were measured in the treated cells by immunofluorescence staining. n>60 cells in (D); n>100 in (E, F). (G) Softening cells reduces cellular contractile forces. Cellular contractility was measured by traction force microscopy. n>18 cells for each condition. (H-J) Stiffening cells has no effect on cell morphology. Huh-7 cells were transfected with CA-MLCK

and CA-ROCK. Cell spreading area and aspect ratio were measured. n>100 cells in (I, J). (K-M) Stiffening cells has no effect on focal adhesion, microtubule, and intermediate filament. n>60 cells in (K); n>100 in (L, M). (N) Stiffening cells enhances cellular contractile forces. n>20 cells for each condition. Scale bar in (A, H): 25 μm. Scale bar in (D-F, K-M): 50 μm. One-way ANOVA was used for the statistical analysis. *, p<0.05; **, p<0.01; ***, p<0.001.

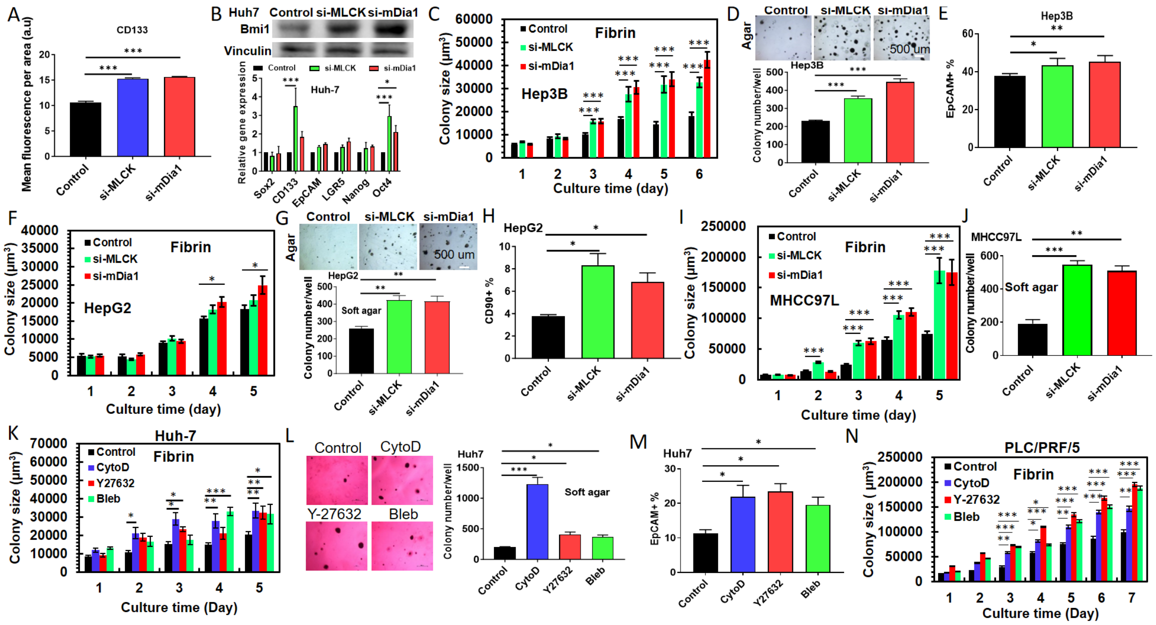

Figure S4 Softening cells enhances tumor cell self-renewal. (A, B) Softening tumor cells up-regulates self-renewal markers. Huh-7 cells were transfected with the siRNAs of MLCK and mDia1. The expressions of CD133 (A), Bmi1, and other self-renewal genes (B) were measured by immunofluorescence staining, western blotting, and qRT-PCR, respectively. n>80 cells in (A) and n=3 in (B). Softening cells promotes the formation and growth of tumor spheroids and the CSC fraction in Hep3B (C-E), HepG2 (F-H), and MHCC97L (I, J). Various HCC cells were transfected with the siRNAs of MLCK and mDia1 and their self-renewal was tested in soft fibrin (C, F, and I) and soft agar (D, G, and J). The fractions of EpCAM+ (E) and CD90+ (H) cells were measured by flow cytometry. n=3. (K-N) Pharmacologically softening

cells enhances self-renewal. The self-renewal of the treated cells was tested in soft fibrin (K, N) and soft agar (L). The fraction of EpCAM+ cells was measured by flow cytometry (M). n=3. One-way ANOVA was used for the statistical analysis. *, $p<0.05$; **, $p<0.01$; ***, $p<0.001$.

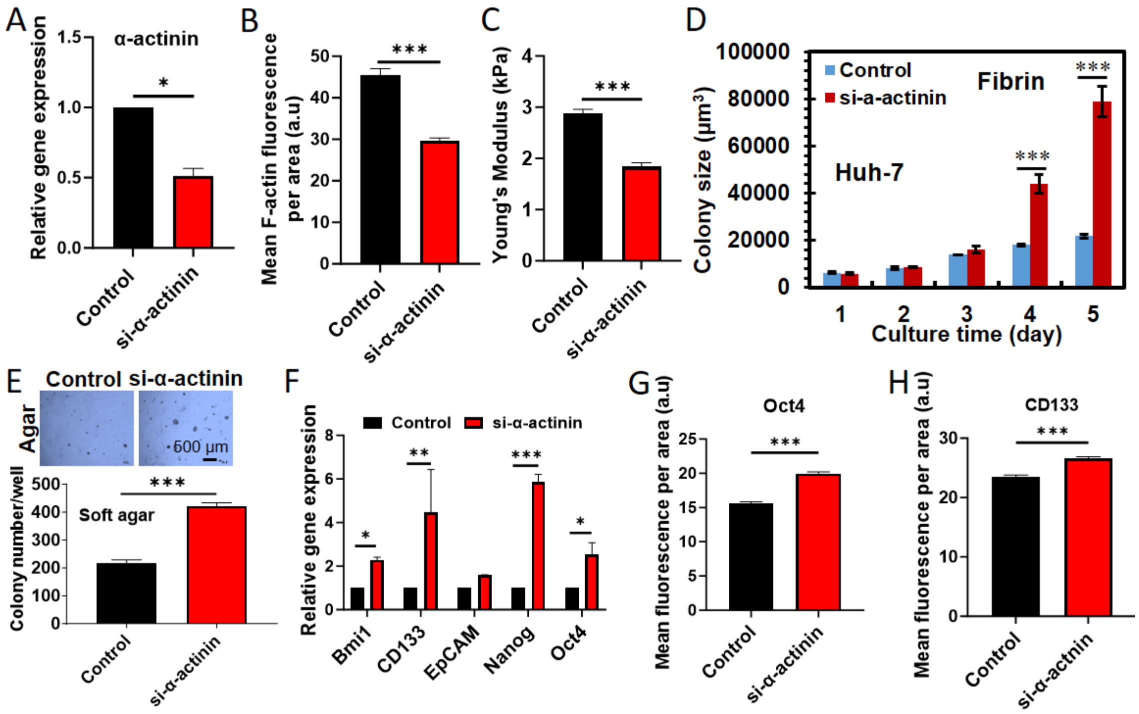

Figure S5 Silencing α-actinin promotes tumor cell self-renewal. (A) The knockdown efficiency of α-actinin siRNA. n=3. (B, C) Silencing α-actinin reduces F-actin and cell stiffness. Huh-7 cells were transfected with α-actinin siRNA. The α-actinin expression was measured by qRT-PCR (A). F-actin was analyzed by phalloidin staining. n>100 cells. The stiffness of these cells was measured by atomic force microscopy. n>120 cells. Silencing α-actinin promotes the growth of tumor spheroids (D), colony formation (E), and the expressions of self-renewal genes (F-H). n=3 in (D-F). n>100 cells in (G, H). Student t-test was used for the statistical analysis. *, $p<0.05$; **, $p<0.01$; ***, $p<0.001$.

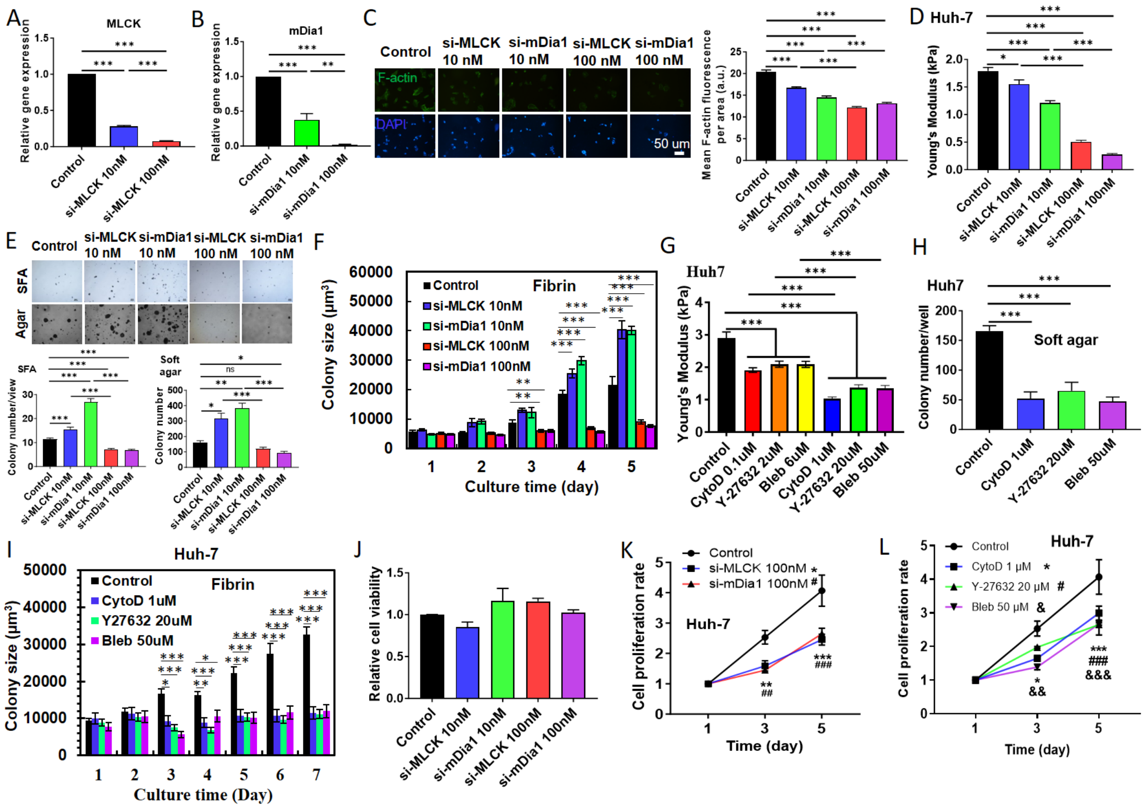

Figure S6 Considerable cell softening suppresses self-renewal. (A, B) The knockdown efficiency of the siRNAs of MLCK and mDia1. n=3. (C, D) High doses of siRNAs further reduce F-actin and cellular stiffness. Huh-7 cells were transfected with 10 nM and 100 nM of MLCK and mDia1 siRNAs, respectively. F-actin and cell stiffness were measured by phalloidin staining and atomic force microscopy, respectively. n>100 in (C); n>70 in (D). Scale bar: 50 µm. (E, F) Moderate/considerable cell softening promotes/suppresses self-renewal. The self-renewal of the treated cells in (C) was tested in soft agar, SFA (E), and soft fibrin (F). n=3. (G) High doses of inhibitors further reduce cellular stiffness. Huh-7 cells were treated with 0.1 and 1 µM Cytochalasin D (CytoD), 2 and 20 µM Y27632, and 6 and 50 µM Blebbistatin (Bleb), respectively. Cell stiffness was measured by atomic force microscopy. n>70. Considerable cell softening suppresses the formation (H) and growth (I) of tumor spheroids. n=3. (J-L) Considerable softening does not affect cell viability but suppresses proliferation. The viability

(J) and proliferation (K, L) of the treated cells in (C, G) were measured at 12 h and the indicated time points by MTS assay. n=3. One-way ANOVA was used for the statistical analysis. *, p<0.05; **, p<0.01; ***, p<0.001.

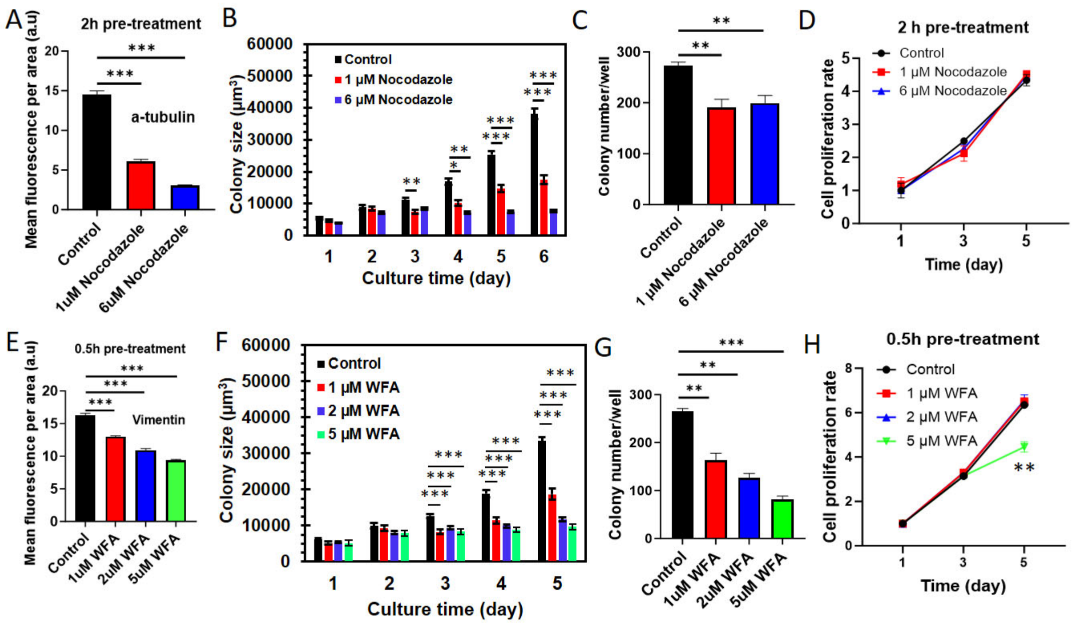

Figure S7 Disrupting microtubule and vimentin suppresses self-renewal. Pharmacologic treatment reduces microtubule (A) and vimentin (E). Huh-7 cells were treated with Nocodazole (1 and 6 μM) and Withaferin A (WFA; 1, 2, and 5 μM), respectively. The expressions of α-tubulin and vimentin were measured by immunofluorescence staining. n>100 cells. (B, C, F, G) Disrupting microtubule and vimentin suppresses the formation and growth of tumor spheroids. The self-renewal of the treated cells in (A, E) was tested in soft fibrin (B, F) and soft agar (C, G). n=3. (D, H) Disrupting microtubule and vimentin has minimal/suppressive effect on cell proliferation. The proliferation of the treated cells was measured at the indicated time points by MTS assay. n=3. One-way ANOVA was used for the statistical analysis. *, p<0.05; **, p<0.01; ***, p<0.001.

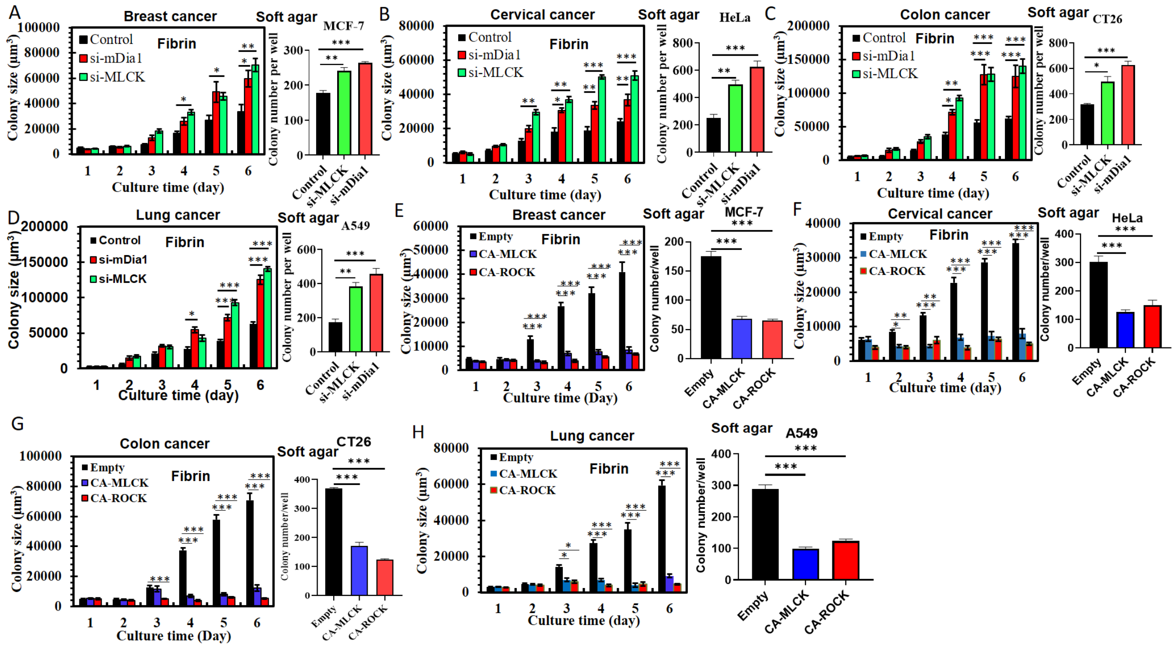

Figure S8 Softening/stiffening cells promotes/suppresses self-renewal in multiple types of cancer. Moderately softening tumor cells enhances self-renewal in breast (MCF-7; A), cervical (HeLa; B), colon (CT26; C), and lung (A549; D) cancer. Tumor cells were transfected with the siRNAs of MLCK and mDia1. The self-renewal of these cells was tested in soft fibrin and soft agar, respectively. Stiffening tumor cells suppresses self-renewal in breast (MCF-7; E), cervical (HeLa; F), colon (CT26; G), and lung (A549; H) cancer. Tumor cells were transfected with CA-MLCK and CA-ROCK plasmids. The self-renewal of these cells was tested in soft fibrin and soft agar, respectively. n=3. One-way ANOVA was used for the statistical analysis. *, $p<0.05$; **, $p<0.01$; ***, $p<0.001$.

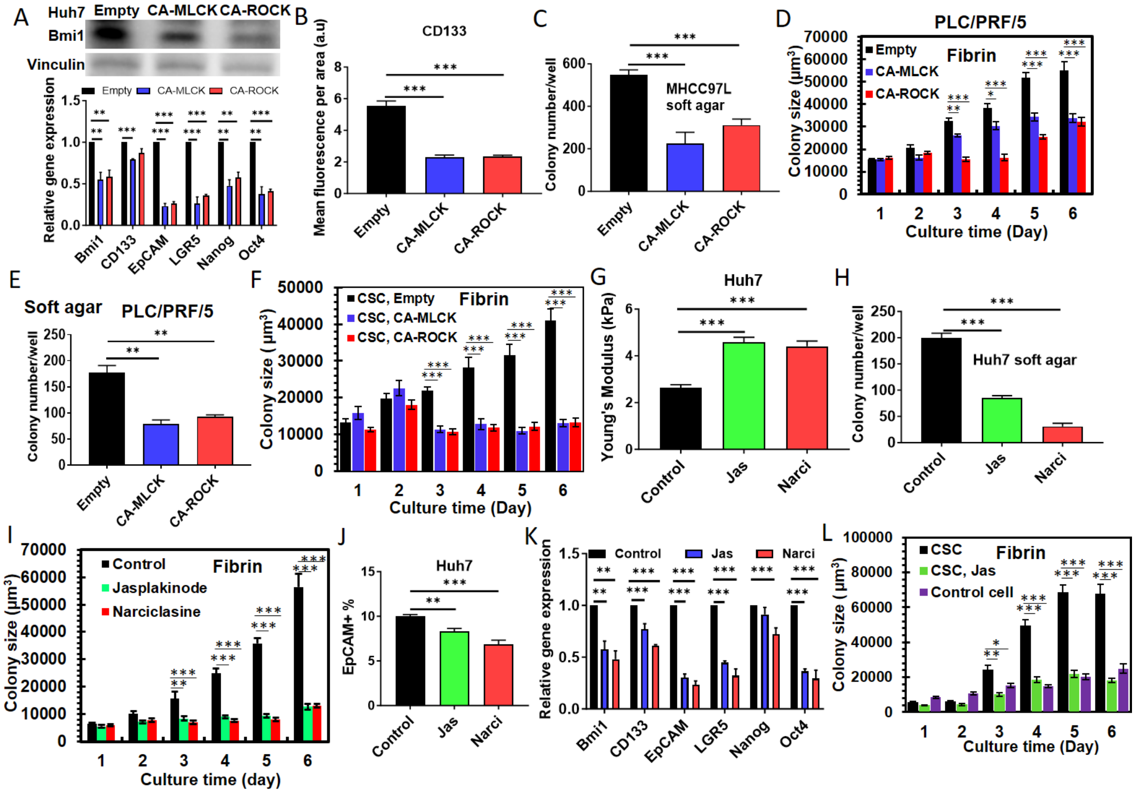

Figure S9 Stiffening cells suppresses self-renewal in multiple types of HCC cells. (A, B) Stiffening cells down-regulates multiple self-renewal genes. Huh-7 were transfected with CA-MLCK and CA-ROCK plasmids. The expressions of the indicated self-renewal genes were measured by western blotting, qRT-PCR (A), and immunofluorescence staining (B). n=3 in (A); n>80 in (B). (C-E) Stiffening cells suppresses self-renewal in multiple HCC cells. MHCC97L (C) and PLC/PRF/5 cells (D, E) were transfected with CA-MLCK and CA-ROCK plasmids. Their self-renewal was tested in soft fibrin and soft agar. n=3. (F) Stiffening CSCs reduces the growth of tumor spheroids in soft fibrin. n=3. (G) Pharmacologic treatment increases cellular stiffness. Huh-7 cells were treated with 20 nM Jasplakinolide (Jas) or 1 nM Narciclasine (Narci). The stiffness of these treated cells was measured by atomic force microscopy. n>110 cells. (H-L) Pharmacologically stiffening HCC cells and CSCs suppresses self-renewal. The self-renewal of the treated cells was tested in soft fibrin and agar (H, I, L). The fraction of EpCAM+ cells was measured by flow cytometry (J). The expressions of

multiple self-renewal genes were quantified by qRT-PCR (K). n=3. One-way ANOVA was used for the statistical analysis. *, $p<0.05$; **, $p<0.01$; ***, $p<0.001$.

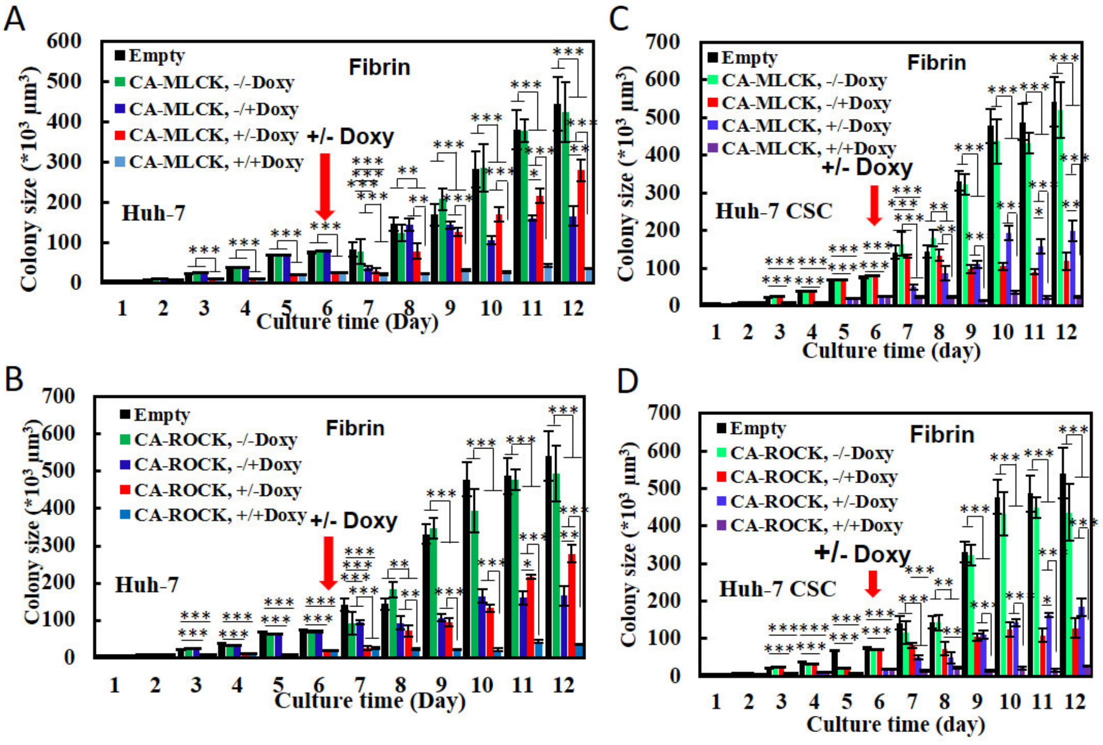

Figure S10 Dynamically modulating tumor cell stiffness regulates their self-renewal in a reversible manner. Dynamic alterations of tumor cell stiffness by activating CA-MLCK and CA-ROCK plasmids influence the growth of tumor spheroids of HCC cells (A, B) and CSCs (C, D) in soft fibrin. Huh-7 cells and CSCs were transfected with CA-MLCK and CA-ROCK plasmids and then cultured in soft fibrin with or without the induction of doxycycline (Doxy). -/-Doxy: without Doxy during the entire period; -/+Doxy: without Doxy during the first 6 days while with Doxy during the last 6 days; +/-Doxy: with Doxy during the first 6 days while without Doxy during the last 6 days; +/+Doxy: with Doxy during the entire period. n=3. One-way ANOVA was used for the statistical analysis. *, $p<0.05$; **, $p<0.01$; ***, $p<0.001$.

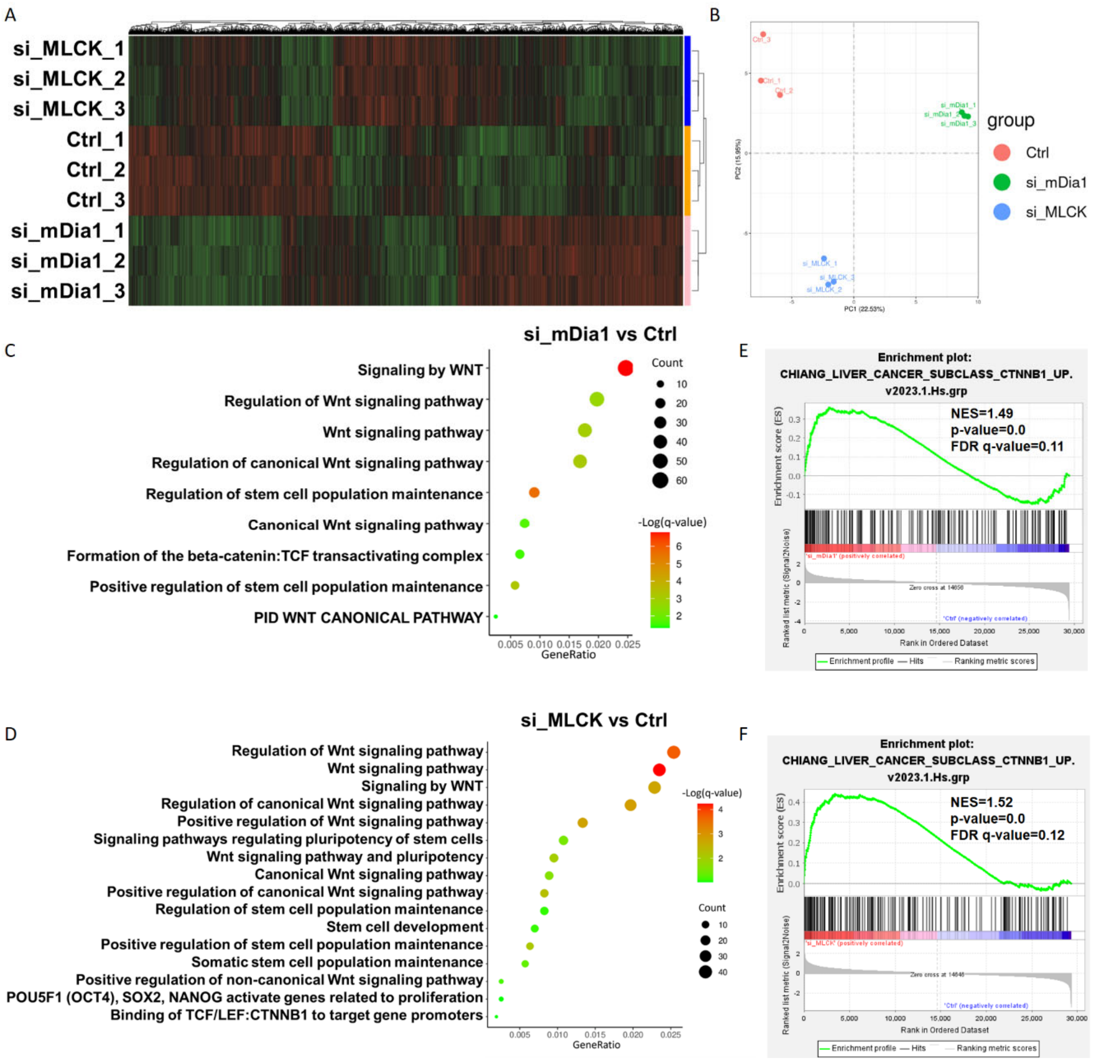

Figure S11 Cell mechanics alter transcriptomic profiles of HCC cells. (A) Heatmap of gene expression of Huh-7 cells in control, si-MLCK, and si-mDia1 groups from RNA-seq analysis. n=3. (B) Principal component analysis (PCA) showing distinct separation of control, si-MLCK, and si-mDia1 groups. (C, D) The Metascape ontology analysis of upregulated genes following knockdown of mDia1 and MLCK in Huh-7 cells. The enriched ontologies related to WNT/β-catenin signaling and stemness were shown. (E, F) GSEA of CHIANG_LIVER_CANCER_SUBCLASS_CTNNB1_UP gene set, as defined by top 200

marker genes up-regulated in CTNNB1 activated HCC. The normalized enrichment scores (NES) and tests of statistical significance (p-value and FDR q-value) were shown.

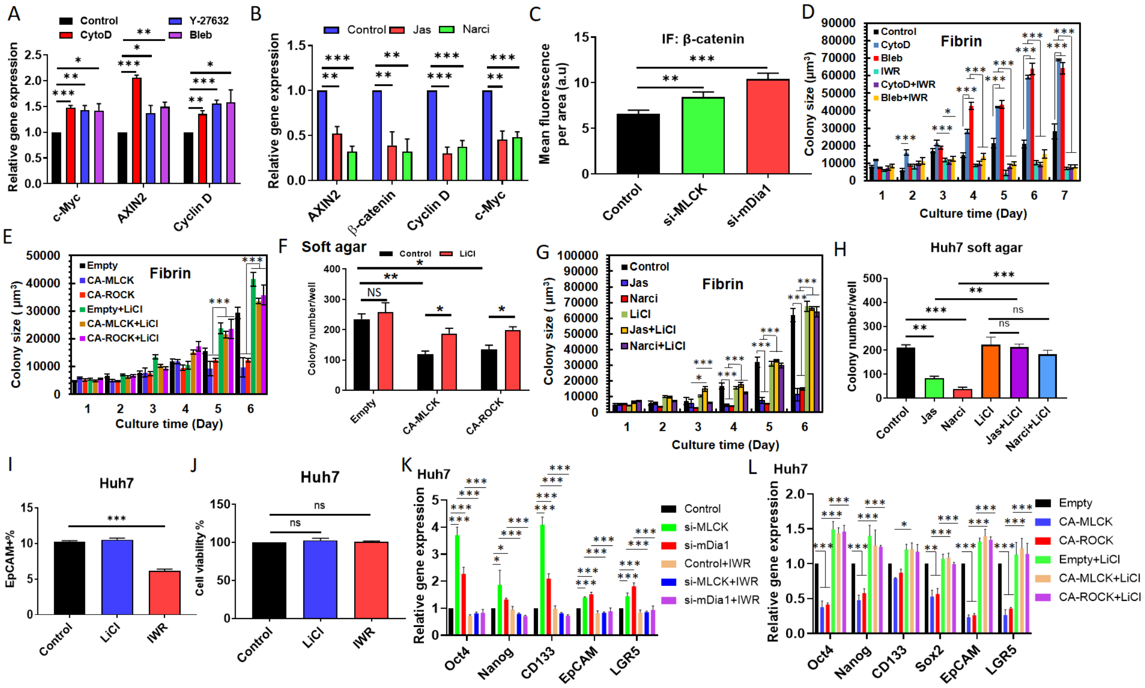

Figure S12 Cell mechanics regulate self-renewal via Wnt signaling. (A, B) Softening/stiffening cells up-/down-regulates the expressions of Wnt downstream genes. Huh-7 cells were treated with 0.1 μM Cytochalasin D (CytoD), 2 μM Y27632, 6 μM Blebbistatin (Bleb), 20 nM Jasplakinolide (Jas), and 1 nM Narciclasine (Narci), respectively. The expressions of the indicated genes were measured by qRT-PCR. n=3. (C) Softening cells increases the expression of β-catenin. Huh-7 cells were transfected with the siRNAs of MLCK and mDia1. β-catenin was measured by immunofluorescence staining. n>30 cells. (D) Inhibiting Wnt signaling diminishes the promotive effect of cell softening on self-renewal. Huh-7 cells were softened with CytoD, Y27632, and Bleb, and then treated with 0.2 μM IWR. These cells were cultured in soft fibrin and the spheroid size was measured. n=3. (E-H)

Activating Wnt signaling rescues the suppressive effect of cell stiffening on self-renewal. Huh-7 cells were stiffened by CA-MLCK and CA-ROCK or Jas and Narci and then treated with 2 μM LiCl. Their self-renewal was tested in soft fibrin (E, G) and soft agar (F, H). n=3. (I) Inhibiting Wnt signaling decreases the fraction of EpCAM+ cells. Huh-7 cells were treated with 0.2 μM IWR and 2 μM LiCl. The fraction of EpCAM+ cells was measured by flow cytometry. n=3. (J) Modulating Wnt signaling has no obvious effect on cell viability. The proliferation of the treated cells was measured at 12 h by MTS assay. n=3. (K, L) Cell mechanics regulate the expressions of self-renewal genes via Wnt signaling. Huh-7 cells were softened/stiffened and then treated with IWR/LiCl. The expressions of the indicated genes were measured by qRT-PCR. n=3. One-way ANOVA was used for the statistical analysis. *, $p<0.05$; **, $p<0.01$; ***, $p<0.001$. ns: no significance.

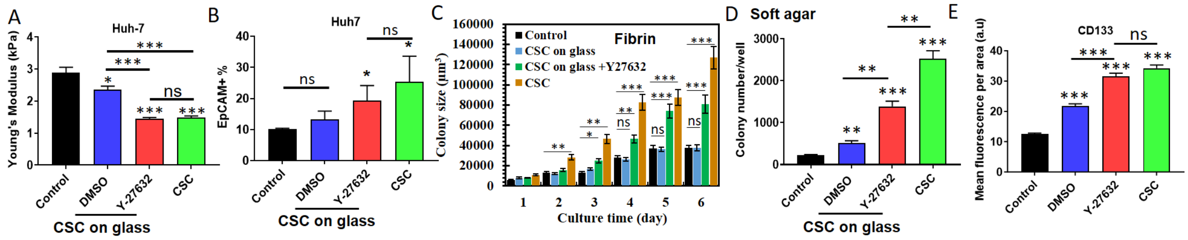

Figure S13 The alteration of cell stiffness is involved in CSC differentiation. (A) HCC CSCs increase cell stiffness after culture on glass. Fibrin-selected CSCs were cultured on glass for 7 days in the presence of Y-27632 or DMSO. The stiffness of these cells was measured by atomic force microscopy. Control: control Huh-7 cells. n>120 cells. (B-E) CSCs become differentiated after culture on glass, which is suppressed by cell softening. The fraction of EpCAM+ cells was measured in the treated cells (B). The self-renewal was tested in soft fibrin (C) and agar (D). The expression of CD133 was analyzed using immunofluorescence staining

(E). n=3 in (B-D); n>80 cells in (E). One-way ANOVA was used for the statistical analysis. *, p<0.05; **, p<0.01; ***, p<0.001. ns: no significance.

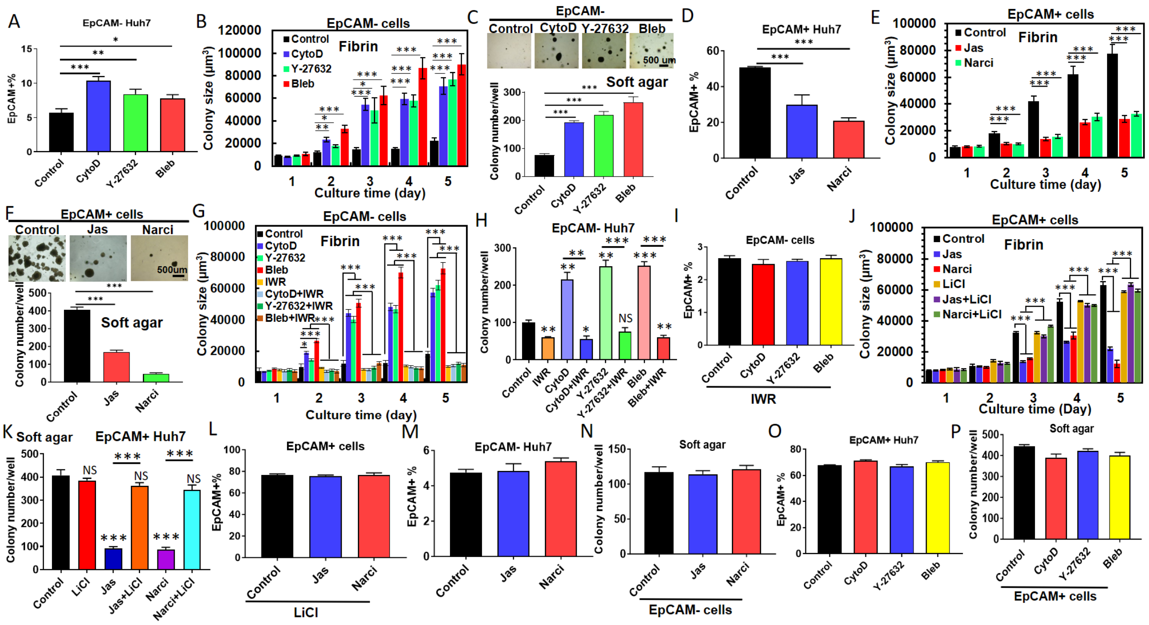

Figure S14 Cell mechanics regulate the interconversion between CSCs and non-CSCs in a Wnt-dependent manner. (A-C) Cell softening facilitates the de-differentiation of EpCAM- cells into EpCAM+ cells. EpCAM- Huh-7 cells were treated with 0.1 μM Cytochalasin D (CytoD), 2 μM Y27632, and 6 μM Blebbistatin (Bleb). The fraction of EpCAM+ cells was measured by flow cytometry (A) and the self-renewal of these treated cells was tested in soft fibrin (B) and agar (C). n=3. (D-F) Cell stiffening facilitates the differentiation of EpCAM+ cells into EpCAM- cells. EpCAM+ cells were treated with 20 nM Jasplakinolide (Jas) and 1 nM Narciclasine (Narci). The fraction of EpCAM+ cells was measured by flow cytometry (D) and the self-renewal of these treated cells was tested in soft fibrin (E) and agar (F). n=3. (G-I) Cell softening mediated de-differentiation of EpCAM- cells into EpCAM+ cells depends on Wnt signaling. EpCAM- cells were treated with 0.1 μM CytoD, 2 μM Y27632, and 6 μM Bleb in the presence or absence of IWR. The self-renewal of these treated cells was tested in soft

fibrin (G) and agar (H). The fraction of EpCAM+ cells was measured when the softened EpCAM- cells were treated with IWR (I). n=3. (J-L) Cell stiffening mediated differentiation of EpCAM+ cells into EpCAM- cells depends on Wnt signaling. EpCAM+ cells were treated with 20 nM Jas and 1 nM Narci in the presence or absence of LiCl. The self-renewal of these treated cells was tested in soft fibrin (J) and agar (K). The fraction of EpCAM+ cells was measured when the stiffened EpCAM+ cells were treated with LiCl (L). n=3. (M, N) Cell stiffening has no suppressive effect on the self-renewal of EpCAM- cells. EpCAM- cells were treated with 20 nM Jas and 1 nM Narci and then used for the analysis of the fraction of EpCAM+ cells (M) and self-renewal in soft agar (N). n=3. (O, P) Cell softening has no promotive effect on the self-renewal of EpCAM+ cells. EpCAM+ cells were treated with 0.1 μM CytoD, 2 μM Y27632, and 6 μM Bleb and then used for the analysis of the fraction of EpCAM+ cells (O) and self-renewal in soft agar (P). n=3. One-way ANOVA was used for the statistical analysis. *, p<0.05; **, p<0.01; ***, p<0.001. ns: no significance.

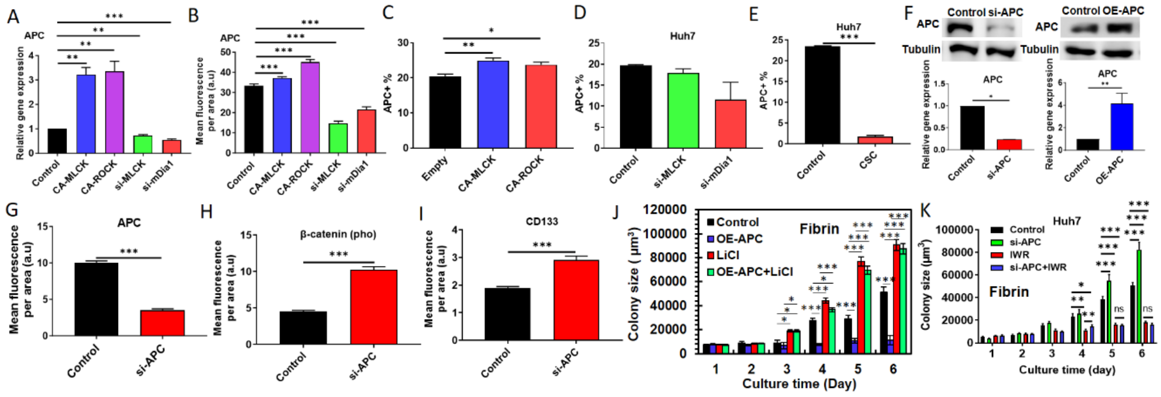

Figure S15 Cell mechanics regulate tumor cell self-renewal through APC/Wnt/β-catenin signaling. (A-D) Cell mechanics affect the expression of APC. Huh-7 cells were transfected with the siRNAs of MLCK and mDia1 or CA-MLCK and CA-ROCK plasmids. The expression of APC was measured at both mRNA (A) and protein levels (B). The fraction of APC+ cells was analyzed by flow cytometry (C, D). n=3 in (A, C, D); n>50 cells in (B). (E) CSCs express

lower level of APC than control cells. n=3. (F, G) Knockdown and overexpression of APC. Huh-7 cells were transfected with APC siRNA or overexpression plasmids (OE-APC). The mRNA (bottom panel in F) and protein (top panel in F; G) expression of APC were measured. n=2 and 3 for the top and bottom panel in F; n>100 cells in (G). (H, I) Silencing APC enhances the phosphorylation of β-catenin and CD133 expression. Huh-7 cells were transfected with APC siRNA and used for the analysis of β-catenin phosphorylation (H) and CD133 expression (I). n>80 cells. (J, K) APC regulates self-renewal through Wnt signaling. Huh-7 cells were transfected with APC siRNA/plasmids and then treated with IWR/LiCl. The self-renewal of these treated cells was assessed in soft fibrin. n=3. One-way ANOVA (A-D, J, K) and student t-test (E-I) were used for the statistical analysis. *, p<0.05; **, p<0.01; ***, p<0.001.

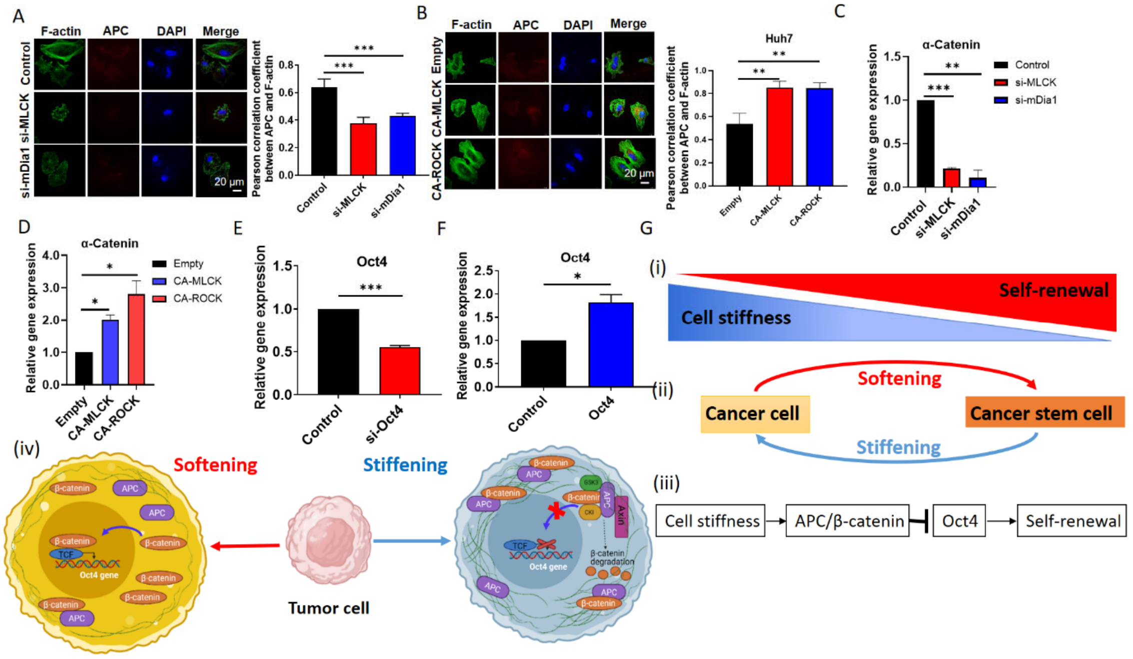

Figure S16 The influence of cell mechanics on the cytoskeleton and self-renewal. (A, B) Softening/stiffening cells decreases/increases the co-localization between APC and F-actin. n>15 cells. (C, D) Cell mechanics influence the expression of α-catenin. n=3. (E, F) Knockdown and overexpression of Oct4. Huh-7 cells were transfected with Oct4 siRNA or

plasmids. The mRNA expression of Oct4 was measured. n=3. (G) The schematic showing the regulatory effect of tumor cell mechanics on self-renewal. One-way ANOVA (A-D) and student t-test (E, F) were used for the statistical analysis. *, $p<0.05$; **, $p<0.01$; ***, $p<0.001$.
